# Artificial Intelligence and Machine Learning for Emergency Department Overcrowding: A Systematic Review with Large Language Model-Assisted Screening

**DOI:** 10.3390/healthcare14172767

**Published:** 2026-09-01

**Authors:** Zekai Wang, Ahmed Qasem, Lin Lu, Bunyamin Ozaydin, Abdulaziz Ahmed

**Affiliations:** 1 Department of Analytics, Charles F. Dolan School of Business, Fairfield University, 1073 North Benson Road, Fairfield, CT 06824, USA; zwang@fairfield.edu (Z.W.); llu@fairfield.edu (L.L.); 2 Department of Health Services Administration, School of Health Professions, The University of Alabama, 1720 2nd Avenue South, Birmingham, AL 35294, USA; aqasem@uab.edu (A.Q.); bozaydin@uab.edu (B.O.)

**Keywords:** emergency department overcrowding, patient flow, length of stay, predictive modeling, large language models, artificial intelligence, machine learning, systematic review

## Abstract

**Background/Objectives**: Emergency department (ED) overcrowding contributes to delayed care, prolonged length of stay (LOS), resource strain, and adverse patient outcomes. This systematic review aimed to examine how artificial intelligence (AI) and machine learning (ML) have been used to address ED crowding and patient flow, with emphasis on modeling approaches, validation practices, and real-world implementation. **Methods**: Following PRISMA 2020 guidelines, Scopus, Embase, Ovid MEDLINE, and CENTRAL were searched for relevant studies published from 2020 onward. After deduplication, 1888 records underwent title and abstract screening using two locally deployed LLaMA models with human adjudication. Screening performance was assessed against 150 manually annotated records. Full-text eligibility assessment and structured data extraction were conducted independently by multiple reviewers, with disagreements resolved by consensus. **Results**: Thirty-two studies were included. Most were retrospective, single-site investigations using electronic health record, administrative, or operational data. Common outcomes included ED LOS, waiting time, occupancy, boarding, disposition, and crowding indices. Tree-based and boosting models frequently performed well, although no approach was consistently superior across tasks and settings. Most studies relied on same-site validation, while external and temporal validation were uncommon. Prospective implementation, workflow integration, model maintenance, and direct operational, clinical, economic, or equity impacts were rarely evaluated. For LLM-assisted screening, LLaMA 4 Scout achieved 84.0% accuracy, 80.0% recall, 88.9% precision, and an F1 score of 84.2%, compared with 78.0%, 67.5%, 88.5%, and 76.6%, respectively, for LLaMA 3.3 on 150 randomly sampled papers. **Conclusions**: AI and ML show promise for addressing ED overcrowding, but the literature remains concentrated at the model-development stage. Future research should prioritize standardized outcomes, multicenter validation, prospective implementation, and direct evaluation of operational and patient-care outcomes.

## 1. Introduction

Emergency department (ED) overcrowding has become a persistent and escalating global healthcare challenge, with significant implications for patient outcomes, operational efficiency, and healthcare system sustainability. Overcrowding occurs when the demand for emergency services exceeds available resources, leading to delays in care, prolonged length of stay (LOS), and increased rates of patients leaving without being seen (LWBS) [1,2]. These operational inefficiencies not only compromise patient safety but are also associated with increased morbidity and mortality, particularly among high-acuity populations [3,4]. In addition, ED overcrowding contributes to staff burnout, resource strain, and broader hospital throughput disruptions, making it a critical issue in both clinical and administrative domains [5].

Over the past decade, there has been growing interest in leveraging artificial intelligence (AI), machine learning (ML), and data science techniques to address ED overcrowding. These approaches enable the analysis of complex, high-dimensional healthcare data and support predictive modeling for key operational outcomes such as patient flow, waiting time, ED occupancy, and LOS. Compared to traditional statistical methods, AI-driven models can better capture nonlinear relationships and temporal dynamics inherent in ED systems, particularly when deep learning architectures are used [6]. Recent studies have demonstrated the potential of these methods to provide real-time decision support, optimize resource allocation, and improve patient throughput.

Despite these advancements, the existing literature remains fragmented and heterogeneous in terms of methodological approaches, evaluation practices, and outcome definitions. Many studies emphasize short-term predictive performance using single-site datasets, with limited consideration of generalizability, external validation, and real-world deployment. In addition, while operational metrics such as LOS and waiting time are frequently examined, broader system-level outcomes, including economic impact, hospital-wide coordination, and patient-centered measures, remain underexplored [7,8]. These limitations highlight the need for a comprehensive systematic review to characterize current AI-, ML-, and data science-driven approaches to ED overcrowding, assess their methodological and practical contributions, and identify priorities for future research.

To address these challenges, this study presents a systematic review of AI-, ML-, and data science-driven approaches for ED overcrowding. Although ED overcrowding was the central focus of this review, we adopted a predefined operational definition encompassing both direct crowding measures and closely related indicators of impaired patient flow. More direct measures included established ED crowding scores such as NEDOCS, occupancy or saturation, and boarding-related measures, whereas related patient-flow measures included waiting time, ED and inpatient LOS, disposition, and other operational outcomes reflecting the movement of patients through the ED and broader hospital system.

The American College of Emergency Physicians (ACEP) has characterized ED overcrowding as a hospital-wide patient-flow problem rather than an isolated ED problem [9,10]. This perspective is supported by evidence that hospital-wide operational bottlenecks, particularly boarding of admitted patients and limitations in inpatient bed availability, can disrupt patient flow, reduce available ED capacity, and contribute to overcrowding [11,12,13,14,15]. Boarding, for example, occurs when admitted patients remain in the ED while awaiting inpatient beds, thereby constraining ED capacity and impeding the flow of other patients through the department. Such bottlenecks may arise from inadequate inpatient bed availability, inefficient discharge planning, and variability in operating room (OR) schedules [16]. The Institute for Healthcare Improvement has emphasized hospital-wide strategies to address ED overcrowding, including improved discharge coordination, proactive inpatient bed management, and smoother OR scheduling [14]. Thus, the broader scope of this review does not imply that all included measures are equivalent indicators of ED overcrowding; rather, it recognizes that overcrowding develops and manifests within an interconnected, hospital-wide patient-flow system.

Recent studies have explored the use of large language models (LLMs) to support systematic review tasks, including study screening, relevance classification, and information extraction [17,18,19,20]. Given the rapid growth and interdisciplinary nature of this literature, we incorporated an LLM-assisted screening pipeline to support efficient title and abstract screening.

Building upon established guidelines such as PRISMA 2020 [21], we combined automated relevance classification with multi-reviewer validation to ensure robust study selection and minimize bias. The objectives of this review are threefold: (1) to systematically characterize the technical and algorithmic foundations of existing approaches; (2) to evaluate the types of outcomes and impacts addressed in the literature; and (3) to assess validation practices, generalizability, and ethical considerations in current research. By providing a structured and comprehensive synthesis, this study aims to inform future research directions and support the development of more effective, scalable, and clinically meaningful AI-driven solutions for ED overcrowding.

The novelty and main contributions of this review can be summarized as follows:

- **Comprehensive methodological comparison:** We systematically compare the included studies in terms of modeling approaches, prediction targets, data sources, feature engineering, validation strategies, performance metrics, and implementation considerations.
- **Coverage of multiple operational scales:** The review considers both patient-level prediction and department-level forecasting applications, providing a broader perspective on how data-driven methods are used to support ED operations.
- **Assessment beyond predictive performance:** In addition to comparing model performance, we examine methodological and implementation issues such as external and prospective validation, model interpretability, generalizability, and translation from predictive accuracy to operational impact.
- **Focus on recent developments:** This review focuses on studies published since 2020, providing an up-to-date synthesis of rapidly evolving applications of AI, machine learning, and data science in emergency department overcrowding and patient-flow management.
- **Identification of research gaps and future directions:** We identify recurring limitations in the current literature and highlight priorities for future research, particularly the need for stronger validation, standardized evaluation and reporting, and greater emphasis on real-world implementation and operational impact.

## 2. Methods

### 2.1. Study Design and Workflow Overview

This systematic review was conducted and reported in accordance with the PRISMA 2020 statement. The review protocol was prospectively registered with “Artificial Intelligence and Machine Learning for Emergency Department Overcrowding: A Systematic Review with Large Language Model-Assisted Screening” and is available at https://osf.io/thz2y/overview?view_only=85b14380a6c343b197e754e06ea0d683 (accessed on 20 August 2026).

**Figure 1:**
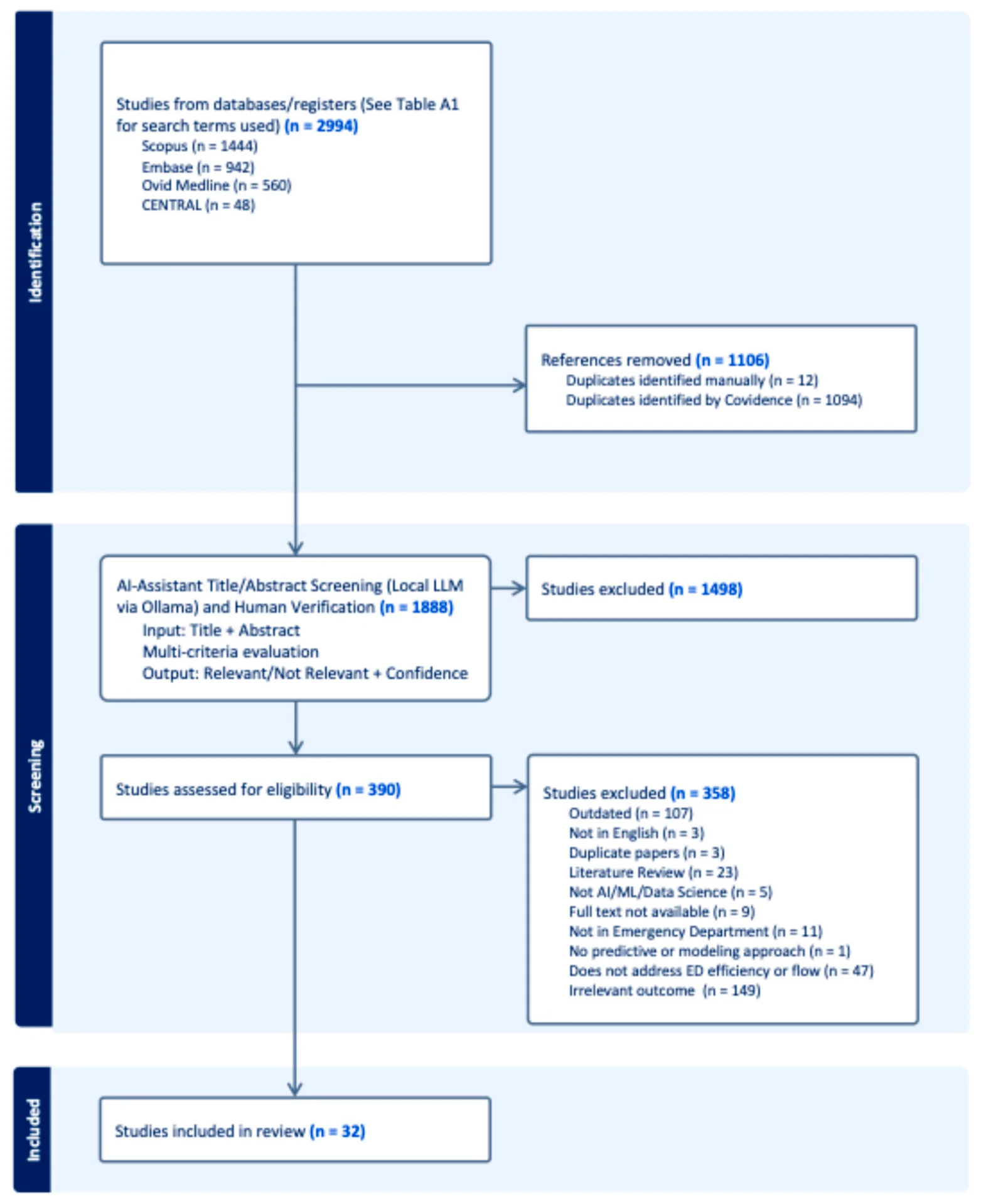
Study Selection Process Following PRISMA Guidelines with AI-Assisted Screening.

This study followed a systematic review protocol in accordance with the PRISMA 2020 guidelines [21]. The overall workflow consisted of four main stages: (1) literature identification, (2) deduplication, (3) LLM-assisted title and abstract screening, and (4) full-text eligibility assessment. Relevant studies published before 2025 were identified through comprehensive searches across multiple bibliographic databases, including Scopus, Embase, Ovid MEDLINE, and CENTRAL. The search strategy was developed around three core concepts: (1) artificial intelligence and data science, (2) hospital emergency services, and (3) emergency department crowding and operational performance. Controlled vocabulary terms were combined with free-text terms searched in titles, abstracts, and keywords. Terms within each concept were combined using the Boolean operator OR, while the three concept groups were combined using AND. The complete database-specific search strategy is provided in Appendix A Table A1. After importing all records into Covidence [22], a web-based software platform designed to streamline and manage the process of conducting systematic reviews, duplicate studies were identified and removed using its built-in deduplication function. The remaining studies were then subjected to a structured screening process. The complete workflow is illustrated in Figure 1.

A total of 2994 records were initially identified from four major databases, including Scopus (*n* = 1444), Embase (*n* = 942), Ovid MEDLINE (*n* = 560), and CENTRAL (*n* = 48). After removing duplicates (*n* = 1106), 1888 unique records were retained for screening.

### 2.2. AI-Assisted Title and Abstract Screening

The title and abstract screening stage was conducted using a hybrid LLM-assisted and human-adjudicated approach. We developed a locally deployed screening pipeline to evaluate all 1888 unique records against the predefined inclusion criteria presented in Table 1.

The screening process was formulated as a multi-criteria decision task. For each study, the LLM was provided with the title and abstract and instructed to evaluate whether the study satisfied three predefined inclusion criteria: (1) the use of AI/ML/data science methods, (2) application in emergency department settings, and (3) relevance to ED operational outcomes such as crowding, efficiency, length of stay, or patient flow (Table 1). A study was classified as relevant only if all three criteria were satisfied.

To operationalize this process, we designed a structured prompt that guided the LLM to perform criterion-level evaluation and evidence extraction in a transparent and reproducible manner. The prompt is shown in Appendix B.

To enhance transparency and interpretability, for each paper, the LLM generated a structured output including: (1) criterion-level evaluation (met/not met), (2) supporting evidence extracted from the abstract, (3) a final decision of *Relevant* or *Not Relevant*, (4) a confidence score ranging from 0% to 100%, and (5) a reasoning summary. The confidence score was not derived from a statistical calculation; rather, it was directly generated by the LLM in response to the prompt instruction: “Then, provide your final decision as ‘RELEVANT’ or ‘NOT RELEVANT’. End with a confidence score from 0–100%.” Accordingly, the score represents the model’s self-assessed confidence in its screening decision. As an additional quality-control measure, records with a confidence score below 80% were flagged for manual human review before the final inclusion or exclusion decision was made.

This structured evaluation framework enabled transparent, auditable, and reproducible decision-making, moving beyond simple binary classification. To enhance reliability and reduce the risk of model bias or hallucination, AI-generated screening decisions were reviewed using a human-in-the-loop process. Human reviewers manually assessed discrepant decisions, low-confidence predictions, and cases flagged as uncertain, and they made the final inclusion or exclusion decisions for these records.

**Figure 2:**
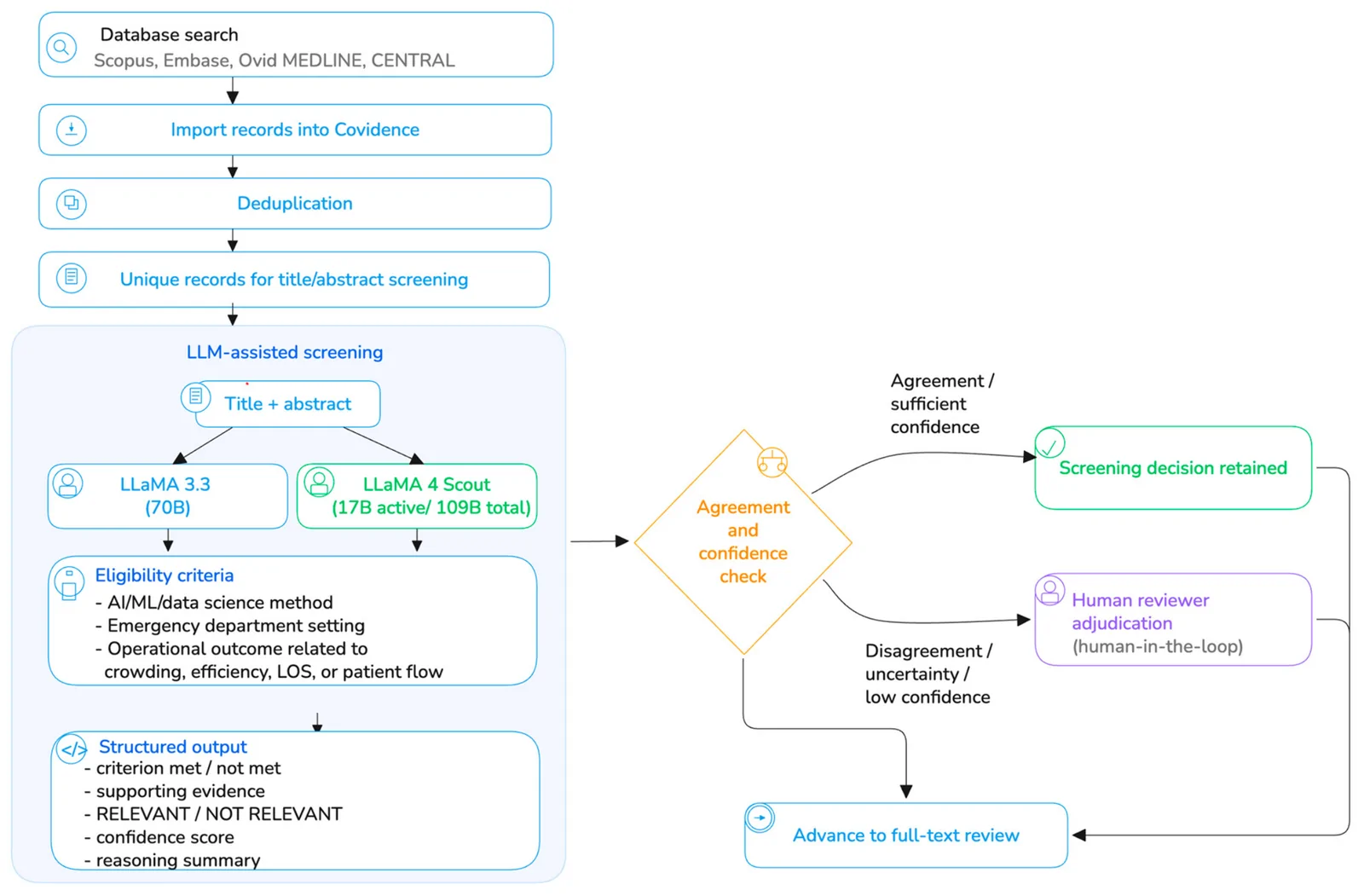
LLM-assisted title and abstract screening workflow.

As illustrated in Figure 2, title and abstract screening was conducted using two LLaMA models: LLaMA 3.3 70B, a 70-billion-parameter dense model, and LLaMA 4 Scout, a mixture-of-experts (MoE) model with 109 billion parameters and 17 billion active parameters [18]. Each model output was entered into Covidence by different human operators to generate initial screening results. To ensure consistency and resolve potential discrepancies, a third human reviewer conducted an independent relevance check on the outputs from both models and adjudicated any conflicts.

To further assess the reliability of the LLM-assisted screening approach, we conducted an additional manual validation experiment. Specifically, 150 papers were randomly sampled from the 1888 records remaining after deduplication and before title and abstract screening. The validation sample was relatively balanced with respect to relevance, with approximately 53% of the papers classified as relevant based on manual review. Each record was manually assessed using the same predefined eligibility criteria applied during the main screening process, and the resulting human screening decisions were treated as the reference standard for validation.

The predictions generated by LLaMA 3.3 and LLaMA 4 Scout were then compared with the corresponding human decisions. Separate confusion matrices were constructed for each model using true positives (TP), false negatives (FN), false positives (FP), and true negatives (TN). Standard classification metrics, including accuracy, recall, precision, and F1 score, were subsequently calculated. Accuracy represents the overall proportion of correctly classified records; recall represents the proportion of truly relevant records correctly identified by the model; precision represents the proportion of records predicted as relevant that were actually relevant; and the F1 score represents the harmonic mean of precision and recall. In addition, 95% confidence intervals (Wilson score interval) were calculated for accuracy, recall, and precision to quantify the uncertainty associated with the performance estimates.

As shown in Table 2, LLaMA 3.3 produced 54 true-positive, 26 false-negative, 7 false-positive, and 63 true-negative decisions, corresponding to an accuracy of 78.0% (95% CI: 70.7–83.9%), recall of 67.5% (95% CI: 56.6–76.8%), precision of 88.5% (95% CI: 78.2–94.3%), and F1 score of 76.6%. LLaMA 4 Scout produced 64 true-positive, 16 false-negative, 8 false-positive, and 62 true-negative decisions, corresponding to an accuracy of 84.0% (95% CI: 77.3–89.0%), recall of 80.0% (95% CI: 70.0–87.3%), precision of 88.9% (95% CI: 79.6–94.3%), and F1 score of 84.2%.

Overall, both models demonstrated relatively high precision, indicating that records classified as relevant were generally consistent with the human screening decisions. LLaMA 4 Scout demonstrated higher accuracy and recall than LLaMA 3.3 and generated fewer false-negative decisions (16 vs. 26), suggesting improved sensitivity for identifying potentially relevant studies. Nevertheless, neither model achieved perfect performance, and both false-positive and false-negative classifications were observed. To address this, we adopted a complementary evaluation strategy by comparing the outputs of both models and incorporating human review to resolve conflicts. Specifically, cases with inconsistent predictions or lower confidence were manually examined to determine the final inclusion decision. This dual-model plus human-in-the-loop approach enhances the robustness and reliability of the screening process, ensuring higher overall accuracy while mitigating potential model errors or biases.

The results show that both LLaMA 3.3 and LLaMA 4 Scout achieve strong and generally consistent performance across all evaluation metrics, indicating their effectiveness in supporting the screening task. While LLaMA 4 Scout demonstrates moderately better performance in terms of accuracy, recall, and F1 score, the overall differences between the two models are not substantial. Importantly, the comparable precision values suggest that both models are similarly effective in limiting false positives, meaning that papers identified as relevant are generally reliable. However, neither model achieves perfect performance, and discrepancies between their predictions were observed. To address this, we adopted a complementary evaluation strategy by comparing the outputs of both models and incorporating human review to resolve conflicts. Specifically, cases with inconsistent predictions or lower confidence were manually examined to determine the final inclusion decision. This dual-model plus human-in-the-loop approach enhances the robustness and reliability of the screening process, ensuring higher overall accuracy while mitigating potential model errors or biases.

Using this LLM-assisted screening pipeline, 1498 studies were excluded at the title and abstract screening stage, and 390 studies were identified as potentially relevant and advanced to full-text review. Overall, this approach significantly reduced the manual screening workload while maintaining methodological rigor and ensuring high-quality study selection.

### 2.3. Full-Text Eligibility Assessment

The 390 studies that passed the initial screening underwent full-text review based on predefined exclusion criteria established prior to analysis. Given the relatively large number of studies remaining after the initial screening, we further focused the review on studies published from 2020 onward to capture the most recent developments and emerging trends in the application of AI, machine learning, and data science to emergency department overcrowding and patient-flow management. This cutoff was considered appropriate because data science and machine-learning methods have evolved rapidly in recent years, including advances in modeling approaches, computational capabilities, data availability, and healthcare implementation. Focusing on studies published since 2020 therefore allowed the review to provide a more current assessment of methodological developments, validation practices, and implementation considerations in this rapidly evolving field.

Studies were excluded for the following reasons: publications prior to 2020 (*n* = 107), non-English studies (*n* = 3), duplicate publications (*n* = 3), literature reviews (*n* = 23), studies not related to AI/ML/data science methods (*n* = 5), full text unavailable (*n* = 9), studies not conducted in emergency department settings (*n* = 11), absence of predictive or modeling components (*n* = 1), studies not addressing emergency department efficiency or patient flow (*n* = 47), and irrelevant outcomes (e.g., studies focused on diagnosis or treatment without connection to ED operations) (*n* = 149).

After full-text screening, a total of 32 studies were included in the final analysis.

### 2.4. Data Extraction and Synthesis Framework

Following full-text eligibility assessment, a total of 32 studies were included in the final analysis. A structured data extraction process was conducted to systematically capture key information from each study. Three reviewers participated in the process, with an overlapping assignment design that ensured that every included study was independently assessed by two different reviewers. Specifically, Reviewers 1 and 2 independently assessed the same 11 studies, Reviewers 1 and 3 independently assessed another 11 studies, and Reviewers 2 and 3 independently assessed the remaining 10 studies. Thus, Reviewer 1 completed 22 assessments, while Reviewers 2 and 3 each completed 21 assessments, resulting in 64 independent assessments across the 32 included studies. Each reviewer first extracted the relevant information independently to minimize the influence of individual judgment. The paired reviewers subsequently compared their extracted results and resolved any discrepancies through discussion until consensus was reached.

The data extraction framework was designed to capture the study, data, analytical, modeling, and evaluation characteristics reported in the included studies. As shown in Table 3, the extracted information was organized into four major domains: (1) study and data characteristics, (2) analytical and prediction characteristics, (3) modeling-task framing and data processing, and (4) model development, validation, performance, and interpretability. Study and data characteristics included the study setting, data sources, sample size, observation period, and level of analysis. Analytical characteristics captured the study aim, overcrowding-related outcome of interest, prediction horizon, and predictor categories. Modeling and data-processing characteristics included the broad analytical approach, task type, target outcome, feature engineering, preprocessing and data-quality procedures, and class-imbalance handling. Model evaluation characteristics covered the models compared, tuning and model-selection procedures, validation strategy, evaluation metrics, best-performing model, and interpretability methods. This structured framework supports a systematic assessment of how AI-, machine learning-, and data science-based approaches to emergency department overcrowding were formulated, developed, validated, and interpreted. Detailed extraction results based on these domains are discussed in the subsequent sections.

## 3. Results

The results are presented in four sections. Section 3.1 describes the general characteristics of the included studies, including publication patterns, study settings, data sources, and overcrowding-related outcomes. Section 3.2 summarizes the technical and algorithmic foundations of the modeling approaches. Section 3.3 synthesizes the reported outcomes and impacts of these models. The final section examines generalizability, reproducibility, and implementation-related considerations. Detailed study-level information is provided in Table 4, Table 5, Table 6 and Table 7.

### 3.1. Study Characteristics

**Figure 3:**
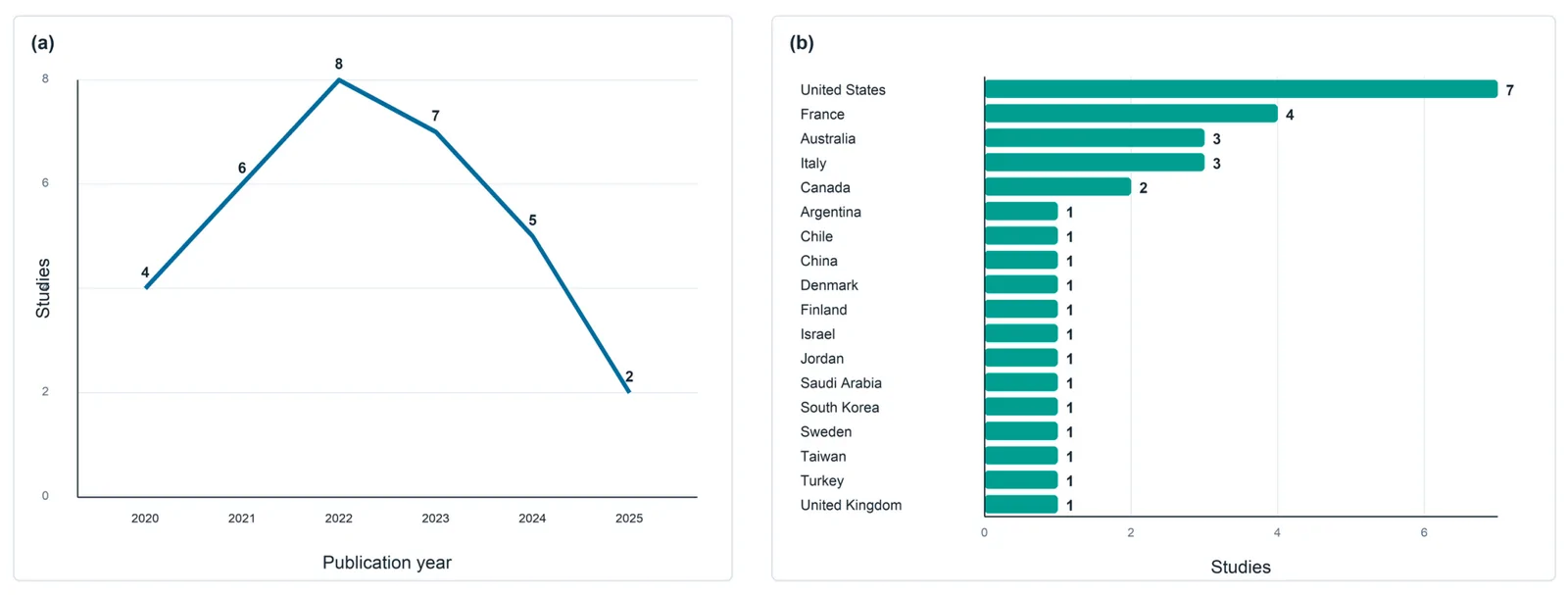
(**a**) Number of included studies by publication year, and (**b**) distribution of studies by country.

A total of 32 studies were included in this review. As shown in Figure 3a, the included studies were published between 2020 and 2025. Publication activity increased notably during the early 2020s and peaked in 2022, and then declined in the most recent years, indicating that research on machine learning for ED overcrowding has expanded substantially in the post-2020 period but remains an evolving field. The geographic distribution of studies was broad but uneven, with studies conducted across North America, Europe, Asia, South America, and the Middle East. The United States contributed the largest share of studies, followed by several European and Asia-Pacific countries, while most other countries were represented by a single study (Figure 3b).

**Figure 4:**
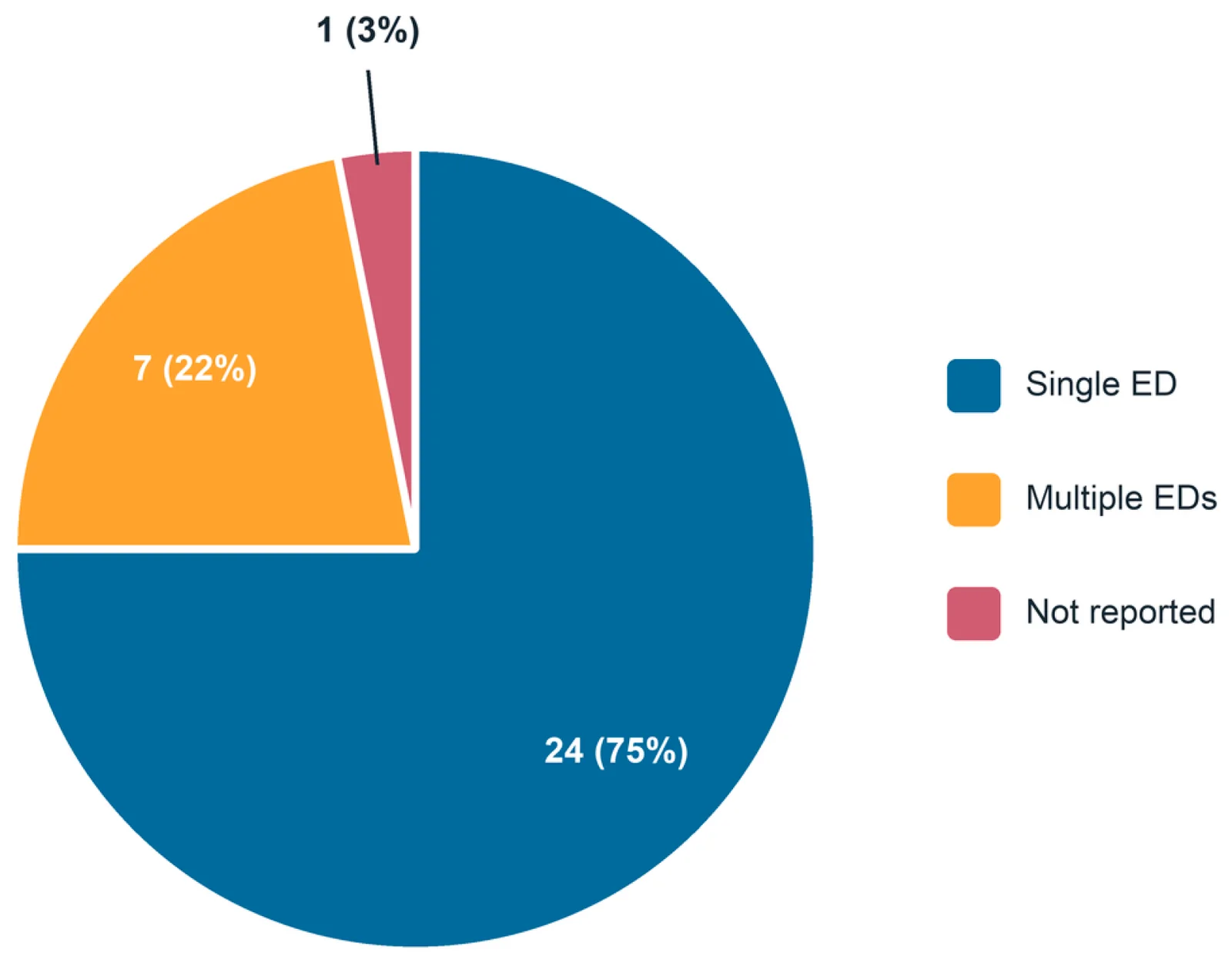
Distribution of included studies by ED setting.

As shown in Figure 4, most studies were conducted in single-ED settings, with 24 studies (75%) using data from one ED, seven studies (22%) using data from multiple EDs, hospital systems, regional systems, or national hospital samples, and one study (3%) not clearly reporting the ED setting. Single-site studies commonly involved academic, tertiary, public, or large urban EDs, although several focused on more specific operational contexts, including pediatric EDs [23], low-acuity patient streams [24,25], hospitalized patients admitted through the ED [26,27,28], and ambulance offload processes [29]. Multisite or broader system-level studies included analyses across multiple EDs or hospitals [30,31,32,33], national hospital-based samples [34], and studies incorporating external or temporal validation across additional ED settings to evaluate cross-site generalizability [35,36].

The included studies were predominantly retrospective observational analyses using routinely collected clinical, administrative, or operational data. Electronic health record (EHR) data, most often combined with administrative records, were the dominant data source and were used in 28 of the 32 studies. Several studies augmented these structured records with auxiliary data streams to capture contextual or environmental influences on ED operations, including weather data [25,36,37,38]; community COVID-19 indicators [32]; emergency medical services (EMS) data [29]; public events, traffic, or web-based signals [38]; and hospital- or population-level administrative datasets [34]. Publicly available and large-scale secondary datasets were also used in a limited number of studies, including MIMIC-IV-ED [39,40], and Hospital Compare linked with American Community Survey and American Hospital Association data [34]. One methodological study used randomly generated synthetic patient journeys rather than real-world clinical records [41]. A smaller subset incorporated unstructured clinical notes [24,26,27,42,43].

Sample size and analytic scale varied considerably across the included studies, reflecting differences in study setting, data structure, and unit of analysis. Patient-level studies commonly analyzed individual ED visits, patient encounters, or admissions and examined outcomes such as ED LOS, waiting time, disposition, and hospital LOS following ED admission. System-level studies, on the other hand, used aggregated operational units, including hourly ED occupancy [44], daily ED aggregates [36], hourly EMS–ED system states [29], hourly/shift crowding states [45], and continuous 4-h aggregated ED states [46]. This variation indicates that machine learning has been applied both to individual patient-flow outcomes and to broader system-level crowding and operational problems. Sample sizes and units of analysis are summarized in Table 4.

The aims of the included studies centered on predicting, explaining, or classifying ED patient-flow and overcrowding-related outcomes (Table 5). ED LOS was the most frequently examined outcome, including delayed LOS [37], prolonged LOS [31], LOS classification [23,40], and LOS in specific groups such as COVID-19 patients [47], low-acuity patients [24], pediatric patients [23]. The second major group of studies focused on waiting time, treatment time, or time to treatment [30,41,48,49]. Other studies addressed more direct crowding measures, including ED occupancy [38], NEDOCS-based overcrowding [36], ED saturation [46], extended boarding and crowding [32], and bed requirements [42].

Prediction horizons varied according to the operational target. Many studies generated predictions at or near the time of ED arrival, registration, triage, admission, or early clinical assessment [30,37,42,50]. Other studies used short-term forecasting horizons, including 1–4 h ahead for ED occupancy [44], 12 h ahead for crowding and boarding-related outcomes [32], and 24-h-ahead ED occupancy forecasting [38]. Several studies did not explicitly report a prediction horizon, particularly those focused on LOS classification, explanatory modeling, or retrospective association analyses [23,39,40,46,51,52].

Across the included studies, predictors generally clustered into three broad domains: patient characteristics, temporal demand patterns, and the operational state of ED and hospital resources (Table 5). Demographic variables were the most common predictor category, appearing in 22 studies, followed by triage or acuity-related variables and temporal or calendar-based variables, each reported in 18 studies. Operational ED-state predictors, such as queue status, occupancy, staffing, or bed availability, were reported in 13 studies and were particularly common in models focused on waiting time, occupancy, or crowding. External predictors were less frequently used: weather data were included in four studies [25,36,37,38], EMS or ambulance-related variables in two studies [29,33], public events, traffic, or web-based signals in one study [38], and broader hospital, community, or population-level characteristics in two studies [32,34]. A smaller group of studies incorporated unstructured clinical text, including triage notes, physician notes, clinical documentation, or free-text event descriptions [24,26,27,42,43].

**Table 4:** Study and data characteristics of the 32 studies included.

Authors	Site(s)	Data Source	Sample Size	Data Period	Level of Analysis
Aldhoayan et al. [37]	Single public ED	EHR (non-clinical) + weather	352,230 visits	Jan. 2017–Oct. 2019	Patient visit
Arnaud et al. [42]	Single academic ED	EHR + admin	105,457 patients	Dec. 2019–Dec. 2021	Patient visit
Arora et al. [35]	Single academic ED; external second-site validation	EHR + admin	352,178/180,715 visits (2 EDs)	2014–2018	Patient visit
Ataman & Sarıyer [48]	Single public ED	EHR + admin	37,711 visits	Aug. 2018	Patient visit
Aziz et al. [39]	Single academic ED	MIMIC-IV-ED	~400,000 visits	2011–2019	Patient visit
Benevento et al. [30]	Two-hospital public ED	EHR + admin	38,081/57,887 visits (2 EDs)	12 month	Patient visit
Chen et al. [24]	Single academic ED	EHR + admin	12,962 low-acuity outpatients	Jan.–Jun. 2017	Patient visit
Cheng et al. [44]	Single academic medical-center ED	EHR + admin	65,132 visits	2012	Hourly ED occupancy
Cheng & Kuo [41]	Not reported	Synthetic (randomly generated)	50,000 patient instances	N/A (simulated)	Patient instance
Chrusciel et al. [26]	Single ED	EHR	5006 stays	Jan.–Sep. 2019	Inpatient stay admitted via ED
Etu et al. [47]	Single academic ED	EHR + admin	3301 COVID-19 patients	Mar.–Dec. 2020	Patient visit
Giunta et al. [36]	Single academic ED; external second-site validation	EHR + admin + weather	730 training days; 365 external-validation days	Jun. 2016–May 2018 (train); 2019 (ext. valid.)	Daily ED aggregate
Gurazada et al. [31]	Three EDs in one hospital	EHR + admin	173,005 visits	2019	Patient visit
Haraldsson et al. [53]	Single academic ED	EHR + admin	49,938 visits	2019	Patient visit
Kadri et al. [23]	Single pediatric ED	EHR + admin	44,676 patients	2011–2012	Pediatric patient visit
Kuo et al. [49]	Single public ED	EHR + admin	12,945 visits	1 month	Patient visit
Li et al. [29]	Single trauma-center ED	EHR + admin	13,486 ambulance calls; 8784 hourly observations	2016	Hourly ED–EMS system state
Moya et al. [43]	Single public ED	EHR + admin	7848 encounters	2018	Patient encounter
Naemi et al. [50]	Single academic ED	EHR + admin + sensors	6027 visits	Jun. 2018–Apr. 2019	Patient visit
Pak et al. [25]	Single public ED	EHR + admin	122,716 visits	2016–2017	Patient visit
Parnass et al. [45]	Single ED	EHR + admin	679,762 visits	Jan. 2013–Jun. 2018	Hourly state and per-shift patient-hours
Rahman et al. [52]	Single public ED	EHR	80,512 visits	2016–2017	Patient visit
Ricciardi et al. [54]	Single academic ED	EHR	496,172 visits	2014–2019	Patient visit
Seo et al. [27]	Single academic ED	EHR + admin	49,266 patients needing hospitalization	Jun. 2018–May 2022	ED patient needing hospitalization
Shbool et al. [51]	Single private ED	EHR	400 patient records	Jan. 2019–Oct. 2020	Patient visit
Smith et al. [32]	Two-hospital ED	EHR + admin + community COVID-19 public data	96,820/42,351 visits (2 EDs)	Pre-COVID Jan. 2019–Feb. 2020; COVID May 2020–Feb. 2021	12-h ED interval
Sulaiman et al. [40]	Single academic ED	MIMIC-IV-ED	410,927 visits	2011–2019	Patient visit
Tomović [33]	Four EDs	EHR + admin	602,646 day-level records	Jan. 2013–Apr. 2018	Daily aggregated ED LOS
Tuominen et al. [38]	Single academic ED	EHR + multiple external sources	210,019 visits	Jan. 2017–Jun. 2019	Hourly ED occupancy
Wang [34]	National hospital ED sample	Hospital Compare, ACS, AHA	11,180 hospital-years (2236 hospitals × 5 y)	2012–2016	Hospital-year aggregate
Wartelle et al. [46]	Single ED	EHR + admin	Not reported	2017–2019	Continuous ED state (4-h aggregated)
Zeleke et al. [28]	Single academic ED	EHR	12,858 admissions (of 84,847 visits)	Jan.–Oct. 2022	Hospitalized ED patient

**Table 5:** Analytical framework and prediction characteristics of the 32 included studies.

Authors	Study Aim	Overcrowding and Patient-Flow Focus	Prediction Horizon	Predictor Categories
Aldhoayan et al. [37]	Identify nonclinical predictors of delayed ED LOS	Delayed ED LOS (>6 h)	At ED admission	Demographics, temporal, weather, ED load
Arnaud et al. [42]	Estimate real-time bed needs and triage support	Patient flow, admission, discharge, bed management	At triage	Demographics, triage, free-text notes, medical history
Arora et al. [35]	Predict individualized ED waiting times	ED waiting time and patient flow	At registration, updated after assessment	Calendar, demographics, staff, ED workload, patient severity
Ataman & Sarıyer [48]	Classify waiting/treatment times and associated factors	ED waiting and treatment times	At registration	Demographics, arrival mode, triage, ICD-10 diagnosis
Aziz et al. [39]	Classify short vs. long ED LOS, derive rules	Extended ED LOS	Not reported	Triage acuity, disposition, comorbidity, transport, chief complaint, age
Benevento et al. [30]	Compare ML techniques for ED patient waiting time prediction	ED waiting time	Real-time at triage	Demographics, temporal, queue-based, arrivals-based, rolling average
Chen et al. [24]	Predict ED LOS using physician notes plus structured data	ED LOS in low-acuity outpatients	Early short-term after first physician encounter	Demographics, vitals, arrival time, triage, physician notes
Cheng et al. [44]	Forecast hourly ED occupancy with time-series models	ED occupancy/crowding	1–4 h ahead	Current occupancy, acuity, boarding, recent arrivals
Cheng & Kuo [41]	Predict 2-h ED wait time; compare LSTM vs. linear regression	ED waiting time	2 h ahead	Patient-pathway timestamps, arrival-time features
Chrusciel et al. [26]	Test whether clinical text improves hospital LOS prediction	Hospital LOS after ED admission	At ED-to-ward transfer	Demographics, ED LOS, triage/diagnosis codes, prior visits, ward, clinical text
Etu et al. [47]	Predict COVID-19 ED LOS delay and related factors	COVID-19 ED LOS delay	At arrival	Demographics, comorbidities, vitals, ED operational factors
Giunta et al. [36]	Develop ED overcrowding models using prior-day data	ED overcrowding (NEDOCS)	1 day ahead	Weather, prior-day crowding, inpatient flow, ED case mix, staffing
Gurazada et al. [31]	Identify factors linked to ED LOS > 4 h; test CDS models	ED LOS > 4 h	At/near ED presentation	Demographics, triage, arrival mode, communication, mental health, admission, diagnostics, doctor wait time
Haraldsson et al. [53]	Model ED LOS and disposition as time-to-event processes	ED LOS and disposition	During ED visit	Demographics, temporal, triage, arrival mode, complaint, diagnosis, imaging, team specialty revisit
Kadri et al. [23]	Predict and classify ED LOS to support decisions	ED LOS	Not reported	Temporal, arrival, demographics, diagnosis, urgency, exams, calendar
Kuo et al. [49]	Deliver real-time personalized waiting-time prediction	ED waiting time	At arrival	Triage, arrival time, staffing, triage/consult/discharge queues
Li et al. [29]	Predict ambulance offload delay for decision support	Ambulance offload delay	1–5 h ahead	Temporal, EMS demand/throughput, NEDOCS, current AOD status
Moya et al. [43]	Predict severity, destination, and LOS from structured and free text	Severity, destination, LOS, hospitalization bottlenecks	At triage, post-diagnosis, and post-hospitalization decision	Demographics, vitals; triage, diagnosis, free-text events
Naemi et al. [50]	Assess how missingness and skewness handling affects ED LOS prediction	ED LOS	At admission	Vital signs, triage, ICD-10 arrival codes, demographics
Pak et al. [25]	Improve waiting-time-to-treatment prediction for low-acuity patients	Waiting time to treatment	After triage	Queue/service flow, rolling averages, time, weather, patient factors, ACU availability
Parnass et al. [45]	Forecast and explain ED crowding with a stochastic model	ED crowding and overcrowding spikes	Hourly/shift crowding distribution	Arrival flux, exit rate, LOS, stochastic noise terms
Rahman et al. [52]	Develop and validate a decision tree for ED LOS > 4 h	ED LOS > 4 h	Not reported	Demographics, triage, consult requests, admission, mental health, temporal/process timing
Ricciardi et al. [54]	Predict ED LOS using a limited set of patient/workflow factors	Prolonged ED LOS	At ED admission	Demographics, triage, arrival mode, admission time
Seo et al. [27]	Predict hospitalization within 24 h and ED waiting time	Hospitalization delay and ED waiting time	Within 24 h of arrival	Demographic, administrative, clinical, free-text variables
Shbool et al. [51]	Identify key factors for ED LOS prediction/classification	ED LOS, waiting time, ED clustering	Not reported	Demographics, temporal, arrival mode, triage, procedures/labs, staffing, crowding, lockdown status
Smith et al. [32]	Forecast ED crowding and assess temporal/spatial data drift	Extended boarding and ARI-related crowding	Next 12 h	Time, patient volume, ED case mix, hospital factors, community factors
Sulaiman et al. [40]	Predict ED LOS and improve interpretability via rule extraction	ED LOS classification	Not reported	Demographics, triage/vitals, comorbidity, chief complaint, disposition, medications, utilization
Tomović [33]	Explain elevated ED LOS and quantify factor impact	LOS elevation	Not applicable	ED visits, ambulance visits, average triage, triage 1–2 count, diversion hours
Tuominen et al. [38]	Forecast 24-h ED occupancy with multivariable inputs	ED occupancy/crowding	24 h ahead	Temporal, weather, bed availability, traffic, public events, web/Google trends, technical indicators
Wang [34]	Estimate average and heterogeneous effects of Medicaid expansion on ED wait time	ED wait time, boarding time, LOS	Not applicable	Hospital and zip-code population characteristics
Wartelle et al. [46]	Measure, forecast, and optimize ED crowding saturation	ED saturation/crowding	Not reported	Arrival/departure flow, patient counts, staffing, service performance
Zeleke et al. [28]	Predict LOS and prolonged LOS after ED admission	Inpatient LOS after ED admission	At ED admission	Demographics, arrival/source; triage acuity, chief complaints

### 3.2. Technical and Algorithmic Characteristics

Table 6 and Table 7 summarize the technical and algorithmic characteristics of the included studies. Overall, the literature was dominated by supervised machine learning approaches applied to classification, regression, and short-term forecasting problems. Most studies addressed ED overcrowding and related patient-flow processes through operational targets such as ED LOS, waiting times, occupancy, admission or disposition, and hospital LOS following ED admission. Classification tasks were common in studies predicting prolonged ED LOS or crowding states, while regression and forecasting approaches were used for continuous waiting-time, LOS, or occupancy prediction. A smaller subset of studies used survival analysis [53], stochastic population modeling [45], and queuing models [46] to represent overcrowding as a dynamic system-level process rather than a conventional patient-level prediction task.

The target outcomes varied substantially across studies, reflecting heterogeneity in how ED overcrowding and related patient-flow processes were operationalized. Aldhoayan et al. [37] and Rahman et al. [52], for example, modeled prolonged ED LOS as a classification task, whereas Zeleke et al. [28] modeled LOS of inpatients admitted through ED as regression and classification problems. Waiting time prediction was another major focus, particularly for real-time operational decision-making, as shown in studies such as [25,35,49]. Other studies focused on more direct measures of system congestion, including ED occupancy [44], NEDOCS-based overcrowding [36], ED saturation [46], and boarding-related crowding [32]. This heterogeneity demonstrates the breadth of ML applications in ED overcrowding research but limits direct comparison of model performance across studies.

Feature engineering was used to convert raw ED data into model-ready predictors (Table 6). Temporal and aggregation-based feature construction was reported in 16 studies, including hourly or daily aggregation, lagged variables, rolling averages, calendar indicators, time-window summaries, and timestamp-derived measures. For example, Giunta et al. [36] derived daily aggregation and prior-day EDOC% measures for overcrowding prediction. Pak et al. [25] incorporated rolling-average features for waiting-time prediction. Encoding and grouping approaches were also reported in 16 studies, including one-hot and ordinal encoding, ICD-10 grouping, and categorical grouping. Several studies further engineered features to represent the operational state of the ED, such as staffing time windows, NEDOCS categories, and prior-day crowding measures. Feature engineering for unstructured data was less frequent, with five studies transforming clinical text into quantitative model inputs. For example, Chen et al. [24] used bag-of-words, TF-IDF, and SVD-derived topics from physician notes, while Chrusciel et al. [26] extracted unified medical language concepts from ED clinical text. Moya-Carvajal et al. [43] incorporated Word2vec embeddings from free-text event descriptions, while Seo et al. [27] used tokenization, bag-of-words, and TF-IDF weighting.

Preprocessing and data-quality handling varied widely across studies. Common steps included missing-value imputation, removal of incomplete or invalid records, outlier detection, feature scaling, and cohort restrictions. Reporting of preprocessing and data-quality procedures varied across studies, with several studies providing limited methodological detail.

The included studies applied a wide range of algorithms (Table 7). Traditional statistical and machine learning models were more common than deep learning and advanced time-series models. Across studies, tree-based and boosting approaches were frequently among the best-performing models, particularly for structured EHR and operational datasets. For example, gradient boosting and related ensemble methods performed well in studies by Etu et al. [47], Seo et al. [27], Sulaiman et al. [40], and Tuominen et al. [38]. However, the best-performing model varied by task, target outcome, feature set, and validation strategy.

Evaluation metrics reflected the diversity of prediction tasks. Classification studies commonly reported accuracy, AUROC, F1-score, precision, and recall. Regression and forecasting studies reported MAE, RMSE, MSE, and MAPE. Although this range of metrics was appropriate for different modeling tasks, inconsistent metric reporting limited cross-study comparison. Calibration, decision-curve analysis, and clinically meaningful error thresholds were reported less frequently than discrimination or prediction-error metrics.

Interpretability were reported in 25 studies, although their depth and form varied considerably. Some studies used inherently interpretable models, such as decision trees [29], regression coefficients [49], and rule-based extraction [39]. Others applied post-hoc explainability methods, including feature importance [35], SHAP [43,53], and calibration plots [28]. However, explainability was not consistently incorporated across studies. When included, it was generally used to identify important predictors rather than to evaluate whether explanations were understandable or useful for ED clinicians and operational leaders.

Clinical deployment and real-world implementation were major gaps. Most studies were retrospective model-development or validation studies and did not report integration into routine ED operations. Few studies proposed future decision-support tools, dashboards, or real-time applications, but these were generally not prospectively evaluated. Arnaud et al. [42] was a notable exception, as their model was deployed through a dashboard to support real-time bed-management decisions during the COVID-19 pandemic. Overall, the literature remains largely at the model-development stage, with limited evidence on implementation feasibility, prospective performance, or operational impact.

**Table 6:** Modeling-task framing and data-processing characteristics of the 32 studies applying ML and AI to ED overcrowding.

Authors	ML/AI Approach	Task Type	Target/Outcome	Feature Engineering	Preprocessing & Data Quality	Class Imbalance Handling
Aldhoayan et al. [37]	ML	Classification	Delayed ED LOS	Grouping; ED-load counts	IQR outlier removal	SMOTE
Arnaud et al. [42]	DL	Classification	ED disposition and required number of beds	One-hot encoding; structured and text inputs	Variable-specific imputation	NR
Arora et al. [35]	ML	Probabilistic forecasting	ED waiting times	Grouping; lags, rolling averages; staff ratios	Incomplete, extreme values removal	NA
Ataman & Sarıyer [48]	Statistical learning	Multi-class classification	ED waiting and treatment times	ICD-10 groups; ordinal recoding	NR	NR
Aziz et al. [39]	Ensemble ML; XAI	Classification	ED LOS class	GB feature importance; PCA; one-hot encoding	Outlier removal; kNN imputation	NR
Benevento et al. [30]	ML	Regression	ED waiting time	Rolling averages; arrival- and queue-based variables	missing, duplicate, outlier removal	NA
Chen et al. [24]	NLP; statistical learning	Regression	ED LOS	BOW/TF-IDF; SVD topics; sclaing	Text cleaning; missing record exclusions	NA
Cheng & Kuo [41]	DL	Regression	ED wait time	Timestamp-difference sequence; scaling	Synthetic uniform generation	NA
Cheng et al. [44]	Time-series forecasting	1–4 h continuous forecasting	Hourly ED occupancy	Weekly seasonality; hourly aggregation	Behavioral-health exclusions	NA
Chrusciel et al. [26]	NLP; ML	Classification	Hospital LOS after ED transfer	One-hot encoding; ICD-10 grouping	UMLS relevance filtering	Median-split balancing
Etu et al. [47]	Ensemble ML	Classification	COVID-19 patients ED LOS	Scaling; Spearman feature selection; one-hot encoding; binning	Incomplete, outlier removal; missing imputation	SMOTE
Giunta et al. [36]	Statistical learning	Classification	ED overcrowding	Daily aggregation; prior-day EDOC%	collinearity and interaction checks	NR
Gurazada et al. [31]	ML	Classification	ED LOS > 4 h	Grouping; nominal encoding	Attribute removal; invalid triage removal	NR
Haraldsson et al. [53]	Survival ML; XAI	Time-to-event analysis	ED LOS	RSF feature selection; survival representation	Outlier removal	NR
Kadri et al. [23]	GAN-driven DL	Regression; LOS class assignment	Pediatric ED LOS	Time-derived features; LOS classes; scaling	Outlier removal; missing imputation	NA
Kuo et al. [49]	ML	Regression	ED waiting time	Staffing time windows; interaction terms; scaling	Imputation; triage-missing and outlier exclusions	NA
Li et al. [29]	ML	Classification	Ambulances on AOD in future	Hourly aggregation; ED queue reconstruction; NEDOCS categorization	Missing values zero-filled; noise removal	NR
Moya et al. [43]	ML; NLP; XAI	Classification; regression	ED Severity; destination; hospital LOS	Word2vec embeddings; dummy variables; PCA; scaling	missing imputation	SMOTE-Tomek
Naemi et al. [50]	ML	Classification; regression	ED LOS at arrival	ICD-10 encoding; one-hot encoding; LOS classes	Multivariate imputation; eligibility exclusions	Borderline-SMOTE; SMOGN
Pak et al. [25]	ML	Regression	Waiting time to treatment	Rolling averages; one-hot encoding	Cohort restrictions	NA
Parnass et al. [45]	Stochastic population model	Crowding forecasting	ED crowding level	Weekly, shift, and hourly aggregation	NR	NA
Rahman et al. [52]	ML	Classification	ED LOS > 4 h	Categorical recoding; time-interval features	Missing values coded as NA	NR
Ricciardi et al. [54]	Ensemble ML	Classification	Prolonged ED LOS	Categorical grouping/encoding	expert-driven feature reduction	3-h threshold to reduce imbalance
Seo et al. [27]	ML; NLP; XAI	Classification; regression	24-h admission and ED waiting time	TF-IDF/BOW; tokenization; scaling	Duplicate removal; missing imputation; text cleaning	NR
Shbool et al. [51]	ML, clustering	Multi-class classification	ED LOS class	EM clustering; correlation ranking	Outlier and incomplete-record exclusion	NR
Smith et al. [32]	ML	Classification	ED crowding	Lag features; scaling	Missing-record exclusion	NR
Sulaiman et al. [40]	Ensemble ML; XAI	Classification	ED LOS class	One-hot encoding; GB feature selection	LOF outlier detection; iterative imputation	SMOTE; Links; ADASYN; Tomek SMOTEENN
Tomović [33]	Instance-based explanation; clustering	Factor-impact explanation	Elevated ED LOS	Daily aggregation; timestamp-derived LOS	NR	NA
Tuominen et al. [38]	Time-series ML; DL	24-h forecasting	Hourly ED occupancy	Past/future covariates; calendar lags; scaling	Exclusions; missing imputation	NA
Wang [34]	Causal ML; econometric modeling	Treatment-effect estimation	ED wait time	Log transform; pre/post averaging; first differences	NR	NA
Wartelle et al. [46]	Queuing model; ML-based forecasting	Regression; simulation-optimization	ED congestion, saturation, departure rate	Time bins; 2-h lags; time-window aggregation	Data anonymization	NA
Zeleke et al. [28]	Ensemble ML	Classification; regression	Hospital LOS after ED admission	One-hot encoding	Excluding transfers, deaths, and incomplete pathways; imputation	Class-weighted RF

**Table 7:** Model comparison, validation, performance, and interpretability characteristics of the 32 studies applying ML and AI to ED overcrowding.

Authors	Models Compared	Tuning & Model Selection	Validation Strategy	Evaluation Metrics	Best Model	Interpretability
Aldhoayan et al. [37]	DT; RF; LogR; ANN; XGB; SVM; NB	Manual	NR	Acc; sens; spec; AUROC	XGB best acc; SVM best AUC	LogR odds ratios
Arnaud et al. [42]	MLP	Threshold strategy	80/20 temporal train-test split	AUROC; acc; sens; F1	AUROC 0.82; acc 86.4%	NR
Arora et al. [35]	RF; quantile regression; quantile LASSO; KNN; LSBoost	Defaults, no tuning	Temporal train-test split; ext validation	RMSE; MAE	QRF best RMSE	RF feature importance
Ataman & Sarıyer [48]	Two LR models	NR	No split	Acc; fit statistics	Treatment LR; 66.4% acc	Coefficients; *p*-values
Aziz et al. [39]	RF; GB	100 estimators; no tuning	80/20 train-test split	Acc; sens; AUROC; rule fidelity	GB; AUROC 72.43%; acc 66.23%	TE2Rules; GB importance
Benevento et al. [30]	LASSO; RF; SVR; ANN; EM	Time-series CV	80/20 train-test split, ext validation	MSE; MAE	EM most accurate; RF best trade-off	Feature-set impact
Chen et al. [24]	Structured LinR; 6 NLP-enhanced LinR	NR	10-fold CV	MSE; MAE	TF-IDF + SVD; MSE 3.00; MAE 1.47	Word clouds; coefficients
Cheng & Kuo [41]	LSTM; LinR	100-fold grid search; SGD; Adam	80/20 train-test split	MAE; SD; 99% CI	LSTM; MAE 28	NR
Cheng et al. [44]	24-SARIMAX; rolling average; Holt-Winters; VAR	MLE order search; AIC	265/100-day train-test split	MSE; MAE; MAPE	24-SARIMAX; MSE 16.20 (1 h)–64.47 (4 h)	NR
Chrusciel et al. [26]	RF (structured & unstructured)	Random search + CV	80/20 train-test split	Acc; recall; F1	Unstructured RF; acc 75.0%; F1 76.4%	RF feature importance
Etu et al. [47]	LogR; GB; DT; RF	10-fold grid search	80/20 temporal train-test split; 10-fold CV	Accuracy; F1; AUROC	GB; acc 85%; F1 0.88; AUC 0.93	DT rules
Giunta et al. [36]	Multivariable LogR	AIC	2/3-1/3 temporal train-test; ext validation	AUROC; calibration slope; PPV; NPV	AUROC 0.997; PPV 95.7%; NPV 98.8%	LogR odds ratios; discrimination plots
Gurazada et al. [31]	ZeroR; J48 DT; RF; KNN; NB; LogR	J48 pruning; KNN k testing	80/20 train-test split	Acc; AUROC; F1	KNN; acc 74.04%; ROC 0.82	J48 rules
Haraldsson et al. [53]	RSF; GB; XGB; DeepSurv	5-fold grid search	5-fold CV	C-index	GB; C-index 0.783	SHAP
Kadri et al. [23]	GAN-P; DBN; CNN; SAE; SVR; RF; AdaBoost; DT	Grid search	Train-test split (ratio NR)	R^2^; RMSE; MAE; MAPE	GAN-P; R^2^ 0.871; RMSE 100.31	NR
Kuo et al. [49]	Stepwise LinR; ANN; SVM; GB	5-fold grid search	70/30 train-test split, 5-fold CV	R^2^; MSE; RMSE	GB; R^2^ 0.429	Coefficients
Li et al. [29]	CART; NB; SVM	CART pruning; no search	10-fold CV; synthetic validation	Acc; sens; spec; F1	NBTree; mean acc 82%	Tree rules; feature importance
Moya et al. [43]	DT; RF; LogR; MLP; LASSO	5-fold grid search	5-fold CV	Acc; F1; R^2^; MSE	DT (severity); MLP (destination); DT (LOS)	SHAP; permutation importance
Naemi et al. [50]	ANN; XGB; RF; SVM; SVR; DT	5-fold grid search	5-fold CV	AUC; AUROC; R^2^; NRMSE	Class. XGB, AUC 0.94; reg. RF R^2^ 0.729	NR
Pak et al. [25]	OLS; ridge; LASSO; RF; quantile regression	k-fold CV with regularization	80/20 temporal train-test split	MSPE; MAPE	LASSO	NR
Parnass et al. [45]	Mean-field deterministic; stochastic Langevin	Two-step MLE estimation	Empirical fitting; no split	R^2^; MSE	Stochastic fit well	Parameter elasticity
Rahman et al. [52]	J48	Pruning	10-fold CV	Acc	J48; acc 84.94%	J48 rules
Ricciardi et al. [54]	RF; MLP; NB; LogR; voting ensemble	Manual adjustment	10-fold CV	Acc; prec; F1	RF; acc 74.88%; prec 72.85%	NR
Seo et al. [27]	RF; MLP; LogR; NB; XGB; LinR	5-fold grid search	80/20 train-test split, 5-fold CV	AUROC; AUPRC; MAE; calibration	XGB; AUROC 0.92; AUPRC 0.68; MAE ~3 h	SHAP; calibration; risk levels
Shbool et al. [51]	LogR; NB; SVM; KNN; REP tree; RF; MLP	MLP learning-rate variants	90/10 temporal train-test split	Acc	REP tree (3-CAT), acc 86.3%; NB best (5-CAT), acc 65.8%	Correlation-based relevance
Smith et al. [32]	LogR; KNN; RF	Regularization	Rolling out-of-sample; temporal ext validation	AUROC	RF; AUC 0.81–0.82	RF feature importance
Sulaiman et al. [40]	GB; RF; LogR; MLP	Defaults; no tuning	80/20 train-test split; 5-fold CV	AUROC; acc; F1	GB; AUC 0.730; acc 69.93%	TE2Rules
Tomović [33]	KNN/clustering impact; RF comparator	User-defined k; Affinity Propagation	Temporal train-test split	Factor-impact proportions; runtime	Clustering clearer/faster; 2.00 s vs. 6.85 s	Per-observation factor ranking
Tuominen et al. [38]	TFT; N-BEATS; DeepAR; LightGBM; ARIMA; HWDM; HWAM	TPE optimization	Temporal split; 365-day backtest	MAE; RMSE; MSIS	LightGBM; MAE 6.63; RMSE 8.77	SHAP
Wang [34]	DiD; causal forest; FDCF	NR	Simulation validation; no split	MSE; adjusted R^2^; *p*-values	FDCF	Heterogeneity plots; subgroup analysis
Wartelle et al. [46]	LogR in Coxian queuing network	NR	Simulation validation; no split	Occupancy relative bias	NA	Congestion metric; departure-rate curves
Zeleke et al. [28]	RF; SVM; GB; AdaBoost; LogR; LinR; LASSO; Ridge; Elastic Net; SVR; XGB	Defaults	70/30 train-test split	AUROC; Brier; F1; RMSE; MAE; calibration	GB best classifier; Ridge/XGB best regressors	GB importance; calibration plots

### 3.3. Outcome & Impact

As shown in Table 6, the reviewed studies predominantly focused on ED overcrowding and closely related patient-flow outcomes, with the majority (22 out of 32) examining throughput-related delays, such as ED LOS [23,24,31,33,37,39,40,47,50,51,52,53,54], waiting times [25,27,30,34,35,41,48,49], and ambulance offload delays [29]. More direct crowding and capacity targets included NEDOCS-based overcrowding [36], ED occupancy [38,44], crowding states [32,45], and ED saturation [46]. On the other hand, economic and resource-related outcomes were largely underrepresented, with most studies not explicitly addressing cost or resource utilization as primary outcomes. Only one study estimated real-time bed requirements based on predicted patient pathways [42], while resource-related factors such as staffing, workload, bed availability, and queue conditions were more often incorporated as model predictors than evaluated as primary outcomes [35,36,38,46,49,51]. Similarly, downstream impacts on broader hospital operations were infrequently evaluated, with only three studies examining hospital LOS after ED admission or transfer [26,28,43]. Finally, clinical and patient-safety outcomes were notably overlooked, with none of the included studies modeling quality-of-care or safety-related measures as primary outcomes.

### 3.4. Evaluation, Validation, and Generalizability

As summarized in Table 7, the reviewed studies predominantly adopted internal validation strategies (27 out of 32). While nine studies incorporated temporal validation (training on earlier data and testing on later data) [25,32,33,35,36,38,42,47,51], a smaller subset number of studies included external validation across different hospitals or regions [30,32,35,36]. Only one study included prospective real-time evaluation [42]. Reproducibility and openness were limited, with 20 studies providing no information on data or code availability, and only a small fraction offering partial access through data sharing or code release. In terms of generalizability, most studies (25 out of 32) did not explicitly evaluate applicability across settings, populations, or temporal conditions, with only a few examining cross-site [30,32,35,36], seasonal, or demand-related variations [32,51], providing limited evidence of generalizability. Interpretability or explanation methods were reported in 25 studies, although explicit consideration of fairness or bias remained very limited. Multimodal modeling was uncommon, with only five studies incorporating unstructured clinical text alongside structured data [24,26,27,42,43].

## 4. Discussion

### 4.1. Principal Findings

The central findings of this review are a gap between predictive performance and demonstrated operational impact. Although the reviewed studies showed that AI and machine learning models can predict a range of ED crowding and patient-flow outcomes, most were at the model-development stage, relied on data from a single ED, and evaluated predictive performance using internal validation. Far fewer studies examined portability across sites or time, prospective implementation, workflow integration, or measurable operational benefit. This pattern suggests that the principal barrier is not the availability of technically capable algorithms, but the difficulty of translating predictions into timely, feasible, and effective operational actions.

The reviewed studies also reveal substantial heterogeneity in how ED overcrowding and patient flow were operationalized. The included studies examined patient-level outcomes, including waiting time, treatment time, ED length of stay, disposition, and admission; department-level outcomes, including occupancy, crowding, congestion, saturation, departure rate, and bed requirements; and cross-boundary outcomes, including ambulance offload delay and hospital length of stay following ED admission or transfer. These targets differ not only statistically but also operationally. Patient-level predictions may support triage, communication, disposition planning, and care-pathway decisions, whereas department-level forecasts may inform staffing, bed management, surge activation, and flow coordination. Their usefulness should therefore be evaluated within the specific decision context and against outcomes that reflect the intended operational action, rather than treating all overcrowding-related outcomes as interchangeable. This heterogeneity limits direct comparison of model performance across studies and highlights the need for standardized, decision-specific definitions and evaluation frameworks.

### 4.2. Data, Modeling, and Operational Relevance

The predictor patterns identified in this review reinforce the view that ED crowding is not solely a patient-level prediction problem, but a dynamic systems problem in which real-time operational context may be at least as important as static patient characteristics. Patient characteristics, including demographics, triage acuity, arrival mode, presenting conditions, and clinical status, may help explain differences in individual waiting time, length of stay, disposition, or admission. However, department-level crowding also depends on real-time operational conditions, such as workload, boarding burden, bed capacity, staffing, hospital occupancy, and prior crowding states. Tree-based and boosting methods frequently performed well on these structured datasets, while deep learning, survival analysis, natural language processing, and stochastic or queuing approaches were used less often. The absence of more advanced and complex algorithms across studies suggests that performance depends more on the alignment among data, target definition, prediction horizon, and workflow use case than on model complexity alone. Algorithm selection should therefore be driven by the operational task and deployment constraints rather than by model complexity alone.

Several studies enriched structured records with contextual information, including weather, traffic patterns, web-based signals, and hospital- or neighborhood-level characteristics. These data sources may help models capture fluctuations in demand and capacity that are not represented in routinely collected ED records. However, richer inputs may also increase preprocessing demands, including missingness imputation, computation burden, and, privacy and governance concerns, while reducing portability when external variables, documentation practices, or information systems differ across institutions. Multimodal models should therefore be evaluated not only for incremental predictive gain, but also for data availability, latency, maintenance burden, and robustness when one or more data streams are missing or delayed.

Importantly, the outcomes assessed in the literature were heavily weighted toward immediate throughput measures. Economic and resource outcomes, downstream hospital effects, clinical safety, patient experience, and equity were rarely primary endpoints. This creates a risk that technically accurate models are judged successful without demonstrating that their use produces meaningful decisions or benefits. For example, predicting a long ED stay has limited operational value unless the prediction is delivered early enough, linked to a feasible intervention, and shown to improve bed allocation, staffing, diagnostics, discharge planning, or escalation.

### 4.3. Model-to-Impact Maturity and the Translational Gap

**Figure 5:**
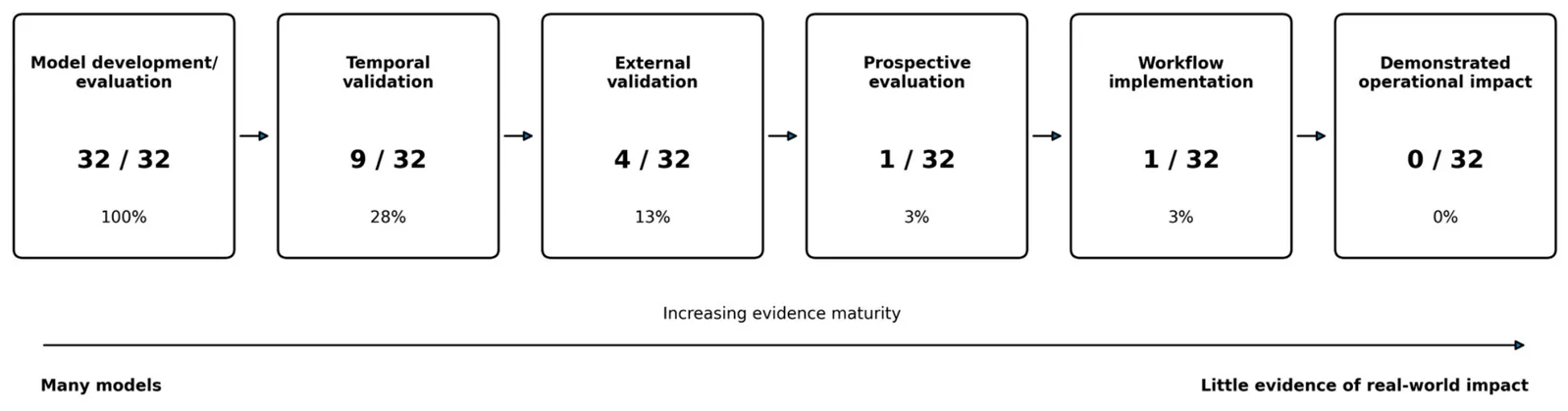
Model-to-impact maturity of AI/DS approaches for emergency department overcrowding, which illustrates how few studies progress from model development to demonstrated real-world operational impact.

Figure 5 summarizes the model-to-impact maturity of the reviewed evidence. The model-to-impact maturity framework distinguishes technical model development from stronger forms of translational evidence. Model development and internal validation establish whether a model can reproduce patterns within a development setting, but they do not establish transportability. Temporal and external validation assess whether performance persists across time and sites, while prospective evaluation determines whether the model functions reliably using real-time data without yet influencing care. Workflow-integrated evaluation then examines usability, alert delivery, response processes, and unintended consequences. The highest level of maturity requires evidence that model-supported actions improve operational, clinical, economic, or equity outcomes.

The reviewed literature was concentrated in the first two stages, with relatively few studies progressing to prospective workflow evaluation or demonstrated impact. Specifically, using the denominator of 32 included studies, all studies reported model development or evaluation, whereas only nine (28%) included temporal validation and four (13%) included external validation. Prospective evaluation and workflow implementation were each reported in only one study (3%), and no study demonstrated a quantified improvement in real-world ED operational outcomes. These stage-specific counts show that the overall pattern is clear: evidence declines sharply as studies move from retrospective model performance toward real-world impact.

This pattern suggests that progress should be judged by more than model performance alone. Internal discrimination or prediction error is necessary, but it is not sufficient evidence of clinical or operational usefulness. Progress toward real-world impact requires advancement through subsequent stages: temporal validation tests resilience to changing demand and practice patterns; external validation tests transportability; prospective silent evaluation reveals performance under live data conditions; workflow implementation assesses usability and adoption; and impact evaluation determines whether the tool changes outcomes. Accordingly, the stages shown in Figure 5 should be viewed as a cumulative evidence pathway rather than as optional extensions of model development.

### 4.4. Validation, Generalizability, Reproducibility, and Governance

The predominance of internal validation is a major limitation of the current evidence. Random splits and conventional cross-validation can produce optimistic estimates when records from similar time periods, care processes, or repeated operational conditions appear in both training and testing data. ED demand, staffing, triage practice, admission policy, and inpatient capacity change over time and vary across institutions. Accordingly, future studies should prioritize temporally ordered holdouts, rolling or back testing designs, external validation across heterogeneous sites, and stress testing during surges or other distribution shifts. Prospective trials should precede deployment to identify data-pipeline failures, latency, calibration drift, and discrepancies between retrospective and live performance.

Model updating and lifecycle management were rarely addressed. A static model may deteriorate as patient populations, documentation, workflows, or capacity constraints change. Deployment studies should predefine monitoring intervals, calibration checks, drift indicators, retraining triggers, and responsibility for approving updates. These technical processes should be embedded within governance structures that define who receives predictions, how actions are documented, who is accountable for overrides, and how safety events are reviewed.

Reproducibility was also limited by incomplete reporting and scarce code or data availability. Patient-level data often cannot be openly shared, but reproducibility can still be improved through publication of code, data dictionaries, feature definitions, prediction-time logic, preprocessing steps, hyperparameter settings, model-selection procedures, calibration results, and executable pipelines using public or synthetic data. Standardized reporting of target definitions, prediction horizons, units of analysis, validation schemes, and implementation context would make comparisons more meaningful and support cumulative evidence development.

Interpretability was more commonly addressed than fairness or accountability. Feature importance, SHAP values, coefficients, rules, and calibration plots can help users understand model behavior, but explanation alone does not establish fairness or safe use. Because historical ED data may encode inequities in access, waiting, triage, admission, language support, and resource allocation, future studies should evaluate discrimination, calibration, error rates, and operational consequences across clinically and socially relevant subgroups. Equity should be assessed in relation to how the model is used, because the same prediction may affect patients differently when it is used to accelerate care, reallocate resources, or lower priority.

### 4.5. LLM-Assisted Screening

This review also provides evidence on the use of locally deployed large language models to assist with title and abstract screening. In the manually annotated sample, LLaMA 4 Scout achieved higher accuracy, recall, and F1 score than LLaMA 3.3, while precision was similar between the two models. Neither model was sufficiently accurate to support autonomous exclusion decisions according to the results in Table 2. For example, the observed recall values (i.e., 67,5% for LLaMA 3.3 and 80% for LLaMA 4 Scout) indicate that autonomous exclusion would have created a meaningful risk of missing eligible studies. The multi-model, human-in-the-loop workflow therefore provided an additional safeguard: structured criterion-level outputs improved auditability, while human reviewers resolved disagreements, uncertainty, and lower-confidence cases. These results demonstrate the feasibility of using locally deployed LLMs as screening aids to support structured screening, but not as replacements for reviewer judgment. Future methodological work should evaluate screening performance across databases and disciplines, report false-negative patterns, model stability, and quantify time savings and reviewer burden.

### 4.6. Future Research Priorities

Future research should move from isolated predictive modeling toward intervention-oriented, lifecycle-based evaluation. Priority studies should: (1) develop and externally validate models using multicenter data that represent diverse EDs and populations; (2) use temporally appropriate validation, calibration assessment, and prospective silent testing; (3) define the operational decision and intervention associated with each prediction; (4) integrate models into existing ED dashboards, command centers, staffing, bed-management, or patient-flow workflows using human-centered design; and (5) evaluate impact through pragmatic trials, stepped-wedge designs, interrupted time-series analyses, or other rigorous quasi-experimental approaches when randomized trials are infeasible.

Evaluation should extend beyond AUROC, accuracy, F1 score, MAE, or RMSE. Studies should measure whether model-supported interventions reduce waiting, LOS, boarding, ambulance offload delay, or patients leaving without being seen; improve staffing and bed utilization; reduce cost and adverse events; improve patient and staff experience; and avoid widening disparities. Researchers should also report alert burden, response time, adoption, overrides, failure modes, and maintenance requirements. Economic evaluation and implementation science frameworks would help determine whether predictive gains justify the infrastructure and organizational change required for deployment.

In summary, AI and machine learning have demonstrated substantial technical potential for modeling ED overcrowding and patient flow, particularly when operational and temporal features are incorporated. The principal challenge is now translational rather than purely algorithmic. Advancing the field will require standardized and decision-specific outcome definitions, stronger temporal and external validation, prospective workflow evaluation, transparent lifecycle governance, and direct evidence that model-enabled actions improve operational, clinical, economic, and equity outcomes.

### 4.7. Limitations of This Review

This review has several limitations. First, the search was restricted to English-language studies published from 2020 onward. This decision focused the review on contemporary AI methods, but it may have excluded relevant earlier work and evidence published in other languages. Second, the included studies were highly heterogeneous in target definitions, analytic units, prediction horizons, datasets, and evaluation metrics; therefore, a quantitative meta-analysis of model performance was not appropriate, and the synthesis is primarily descriptive. Third, although this study demonstrates the feasibility of using LLMs to support structured screening, it did not directly quantify time savings, reviewer workload, or cost. These potential efficiency benefits should therefore be evaluated in future studies rather than assumed. In addition, although the use of two LLMs and additional human review of low-confidence cases was intended to improve screening reliability, concordant decisions between the models do not necessarily guarantee correctness. In particular, both models could potentially assign a high-confidence *Not Relevant* decision to a truly relevant study, resulting in a concordant false negative that would not automatically trigger human review. Manual validation of a randomly selected subset of records helped assess the overall reliability of the screening process, but this possibility cannot be completely excluded and should be considered a limitation of the LLM-assisted screening framework.

## 5. Conclusions

This systematic review synthesized 32 studies applying artificial intelligence, machine learning, and data science methods to emergency department overcrowding and related patient-flow challenges. The included studies examined a wide range of outcomes, including ED length of stay, waiting time, occupancy, crowding, boarding, ambulance offload delay, disposition, and downstream hospital length of stay. Tree-based and boosting models often performed well on structured ED data, but no modeling approach was consistently superior across different outcomes, prediction horizons, datasets, and operational settings.

Despite encouraging technical results, most studies remained retrospective, single-site, and focused on internally validated predictive performance. External and temporal validation were limited, prospective evaluations and workflow-integrated implementations were rare, and direct evidence of improved operational, clinical, economic, or equity outcomes was largely absent. These findings indicate that the principal challenge facing the field is no longer model development alone, but the translation of predictive outputs into timely, feasible, and beneficial operational actions.

The LLM-assisted screening process used in this review also demonstrated the potential of locally deployed language models to improve the efficiency of evidence synthesis. Nevertheless, imperfect recall and disagreement between models underscore that LLMs should be used as assistive rather than autonomous screening tools. Transparent criterion-level outputs, confidence assessment, and human adjudication remain essential for maintaining methodological rigor and minimizing erroneous study exclusions.

Future studies should use multicenter and longitudinal datasets, standardized and decision-specific definitions of overcrowding outcomes, stronger temporal and external validation, prospective testing, and workflow-based impact evaluation. They should also assess whether model-supported interventions reduce waiting, boarding, length of stay, ambulance offload delay, or patients leaving without being seen, while improving resource use, patient safety, staff experience, and equity. Demonstrating real-world operational benefit, rather than predictive performance alone, will be essential for the meaningful and sustainable use of AI and machine learning algorithms in ED overcrowding management.

## Figures and Tables

**Table 1:** Predefined Inclusion Criteria for AI-Assisted Screening.

Criterion	Description	Operational Definition in LLM Evaluation
Criterion 1	Use of AI/ML/Data Science methods	The study explicitly discusses artificial intelligence, machine learning, or data science techniques, including predictive models, deep learning, or large language models
Criterion 2	Emergency Department (ED) setting	The study is conducted in or applied to emergency department, emergency service, or emergency room settings
Criterion 3	ED operational outcomes	The study addresses ED-related operational outcomes such as crowding, efficiency, length of stay, or patient flow

**Table 2:** Confusion Matrices and Performance of LLaMA 3.3 and LLaMA 4 Scout Against Human Screening Decisions in the 150-Record Validation Sample.

Model	TP	FN	FP	TN	Accuracy (95% CI)	Recall (95% CI)	Precision (95% CI)	F1
LLaMA 3.3	54	26	7	63	78.0% (70.7–83.9%)	67.5% (56.6–76.8%)	88.5% (78.2–94.3%)	76.6%
LLaMA 4 Scout	64	16	8	62	84.0% (77.3–89.0%)	80.0% (70.0–87.3%)	88.9% (79.6–94.3%)	84.2%

**Table 3:** Data Extraction Framework for Included Studies.

Domain	Category	Description
Study and Data Characteristics	Study identification	Authors and reference number
	Study setting and sites	Type and number of EDs, hospitals, or healthcare settings included
	Data sources	Origin and type of data
	Sample size	Number of patients, observations, or simulated instances analyzed
	Data period	Calendar period or simulated timeframe represented in the dataset
	Level of analysis	Unit at which observations were analyzed
Analytical and Prediction Characteristics	Study aim	Primary analytical, predictive, or decision-support objective
	Overcrowding and patient-flow focus	ED overcrowding or related patient-flow construct examined
	Prediction horizon	Time at which predictions were generated or the future period being predicted
	Predictor categories	Major groups of input variables
Modeling-Task Framing and Data Processing	AI/ML/DS approach	Broad analytical approach
	Task type	Modeling formulation
	Target or outcome	Specific variable, event, or operational state predicted, explained, or optimized
	Feature engineering	Variable construction and representation methods
	Preprocessing and data quality	Procedures for addressing data-quality issues
	Class-imbalance handling	Approaches used to address imbalanced outcomes; recorded as not applicable for no classification tasks
Model Development, Validation, Performance, and Interpretability	Models compared	Models evaluated within each study
	Tuning and model selection	Hyperparameter tuning, model selection, regularization, threshold optimization, or default settings
	Validation strategy	Internal, temporal, external, cross-validation, rolling out-of-sample, back testing, synthetic, simulation-based, or empirical validation procedures
	Evaluation metrics	Measures of discrimination, classification performance, prediction error, model fit, or simulation accuracy
	Best-performing model	Model or approach identified as providing the strongest performance
	Interpretability	Methods used to explain model behavior or predictor contributions

## Data Availability

No new data were created or analyzed in this study.

## Abbreviations

The following abbreviations are used in this manuscript:

Acc	accuracy
ACS	American Community Survey
ADASYN	adaptive synthetic sampling
admin	administrative data
AdaBoost	adaptive boosting
AHA	American Hospital Association
AIC	Akaike information criterion
AOD	ambulance offload delay
ARI	acute respiratory illness
ARIMA	autoregressive integrated moving average
AUPRC	area under the precision–recall curve
AUROC	area under the receiver operating characteristic curve
BOW	bag-of-words
CART	classification and regression tree
CI	confidence interval
CNN	convolutional neural network
CV	cross-validation
DBN	deep belief network
DeepAR	deep autoregressive model
DiD	difference-in-differences
DL	deep learning
DT	decision tree
DTFP	door-to-first-provider time
ED	emergency department
EDOC	emergency department occupancy
EDOC%	emergency department overcrowding percentage
EHR	electronic health record
EM	expectation–maximization
EMS	emergency medical services
ext	external
FDCF	factor-driven causal forest
GAN	generative adversarial network
GAN-P	generative adversarial network predictor
GB	gradient boosting
HWAM	Holt–Winters additive model
HWDM	Holt–Winters damped model
ICD-10	International Classification of Diseases, 10th Revision
IQR	interquartile range
kNN	k-nearest neighbors
LASSO	least absolute shrinkage and selection operator
LBTC	left before treatment completion
LightGBM	light gradient-boosting machine
LinR	linear regression
LOF	local outlier factor
LogR	logistic regression
LOS	length of stay
LSBoost	least-squares boosting
LSTM	long short-term memory
LWBS	left without being seen
MAE	mean absolute error
MAPE	mean absolute percentage error
MIMIC-IV-ED	Medical Information Mart for Intensive Care IV—Emergency Department module
MLE	maximum likelihood estimation
ML	machine learning
MLP	multilayer perceptron
MSE	mean squared error
MSIS	mean scaled interval score
MSPE	mean squared prediction error
N/A	not applicable
N-BEATS	neural basis expansion analysis for interpretable time-series
NB	naïve Bayes
NEDOCS	National Emergency Department Overcrowding Scale
NLP	natural language processing
NPV	negative predictive value
NR	not reported
NRMSE	normalized root mean squared error
PCA	principal component analysis
PPV	positive predictive value
R^2^	coefficient of determination
RF	random forest
RMSE	root mean squared error
RSF	random survival forest
SAE	stacked autoencoder
SARIMAX	seasonal autoregressive integrated moving average with exogenous variables
SD	standard deviation
sens	sensitivity
SGD	stochastic gradient descent
SHAP	Shapley additive explanations
SMOGN	synthetic minority oversampling technique for regression with Gaussian noise
SMOTE	synthetic minority oversampling technique
SMOTEENN	SMOTE with edited nearest neighbors
spec	specificity
SVD	singular value decomposition
SVM	support vector machine
SVR	support vector regression
TE2Rules	tree ensemble to rules
TF-IDF	term frequency–inverse document frequency
TFT	temporal fusion transformer
TPE	tree-structured Parzen estimator
UMLS	Unified Medical Language System
VAR	vector autoregression
XAI	explainable artificial intelligence
XGB	extreme gradient boosting

## Appendix A. Search Strategy

**Table A1:** Summary of the Search Strategy.

Search Concept	Controlled Vocabulary Terms	Free-Text Terms
Artificial intelligence and data science	exp Artificial Intelligence/; exp Data Science/	predict* adj (analysis or model* or method* or learn*); “artificial intelligence”; machine-learn*; “deep learning”; “data mining”; “Large Language Model”; reinforcement-learning*; ensemble-learn*; ensemble-model-aggregation*; federated-learn*; learning-from-labeled-data; Chat-GPT; ChatGPT*; natural-language-process*; neural-network*; perceptron*
Hospital emergency services	exp Emergency Service, Hospital/	(emergency or trauma) adj2 (room* or ward* or department* or service* or unit* or center* or centre* or area* or care or medicine); Emergicenter*
Crowding and operational performance	exp Crowding/; exp Length of Stay/; exp Workload/; exp Efficiency, Organizational/; exp Admitting Department, Hospital/	(Efficiency or Effectiveness).ti.; Crowd*; congest*; overcrowd*; caseload*; workload*; Patient adj (flow or throughput or turnover); length* adj3 (stay* or hospital*); (case or work) adj load*; (Department or clinic) adj volume; Wait* adj (time* or list*); Time adj2 treatment

Note: exp indicates that a controlled-vocabulary term was exploded to include all narrower terms in the database hierarchy; * indicates truncation; adj, adj2, and adj3 indicate proximity operators; .ti indicate title.

## Appendix B. Prompt Used for LLM-Assisted Title and Abstract Screening

You are a research assistant evaluating the relevance of academic papers based on specific search criteria. The search criteria focuses on papers that discuss: 1. Artificial Intelligence, Machine Learning, or Data Science approaches (including predictive models, deep learning, LLMs, etc.) 2. Applied in Emergency Department/Service/Room settings 3. Addressing issues of crowding, efficiency, length of stay, or patient flow Here is the paper information: Title: {title} Abstract: {abstract} Determine if this paper meets ALL THREE criteria: 1. Does it discuss AI/ML/Data Science methods? 2. Is it applied in emergency department settings? 3. Does it address efficiency, crowding, length of stay, or patient flow? First, analyze each criterion separately with specific evidence from the abstract. Then, provide your final decision as “RELEVANT” or “NOT RELEVANT”. End with a confidence score from 0–100%. Your output must be in the following JSON format: { “criterion1_met”: true/false, “criterion1_evidence”: “Quote evidence from the abstract”, “criterion2_met”: true/false, “criterion2_evidence”: “Quote evidence from the abstract”, “criterion3_met”: true/false, “criterion3_evidence”: “Quote evidence from the abstract”, “decision”: “RELEVANT" or "NOT RELEVANT”, “confidence”: 85, “reasoning”: “Brief explanation of your decision” }

## Appendix C. PRISMA Checklist

**Table A2:** PRISMA Checklist.

Section and Topic	Item #	Checklist Item	Location Where Item Is Reported
**TITLE**			
Title	1	Identify the report as a systematic review.	1
**ABSTRACT**			
Abstract	2	See the PRISMA 2020 for Abstracts checklist.	1
**INTRODUCTION**			
Rationale	3	Describe the rationale for the review in the context of existing knowledge.	2
Objectives	4	Provide an explicit statement of the objective(s) or question(s) the review addresses.	2–3
**METHODS**			
Eligibility criteria	5	Specify the inclusion and exclusion criteria for the review and how studies were grouped for the syntheses.	3
Information sources	6	Specify all databases, registers, websites, organisations, reference lists and other sources searched or consulted to identify studies. Specify the date when each source was last searched or consulted.	3
Search strategy	7	Present the full search strategies for all databases, registers and websites, including any filters and limits used.	3
Selection process	8	Specify the methods used to decide whether a study met the inclusion criteria of the review, including how many reviewers screened each record and each report retrieved, whether they worked independently, and if applicable, details of automation tools used in the process.	3–6
Data collection process	9	Specify the methods used to collect data from reports, including how many reviewers collected data from each report, whether they worked independently, any processes for obtaining or confirming data from study investigators, and if applicable, details of automation tools used in the process.	6–8
Data items	10a	List and define all outcomes for which data were sought. Specify whether all results that were compatible with each outcome domain in each study were sought (e.g., for all measures, time points, analyses), and if not, the methods used to decide which results to collect.	6–8
	10b	List and define all other variables for which data were sought (e.g., participant and intervention characteristics, funding sources). Describe any assumptions made about any missing or unclear information.	6–8
Study risk of bias assessment	11	Specify the methods used to assess risk of bias in the included studies, including details of the tool(s) used, how many reviewers assessed each study and whether they worked independently, and if applicable, details of automation tools used in the process.	5–6
Effect measures	12	Specify for each outcome the effect measure(s) (e.g., risk ratio, mean difference) used in the synthesis or presentation of results.	n.a.
Synthesis methods	13a	Describe the processes used to decide which studies were eligible for each synthesis (e.g., tabulating the study intervention characteristics and comparing against the planned groups for each synthesis (item #5)).	7–8
	13b	Describe any sensitivity analyses conducted to assess robustness of the synthesized results.	5–6
Reporting bias assessment	14	Describe any methods used to assess risk of bias due to missing results in a synthesis (arising from reporting biases).	n.a.
Certainty assessment	15	Describe any methods used to assess certainty (or confidence) in the body of evidence for an outcome.	5
**RESULTS**			
Study selection	16	Describe the results of the search and selection process, from the number of records identified in the search to the number of studies included in the review, ideally using a flow diagram.	4
Study characteristics	17	Cite each included study and present its characteristics.	8–15
Risk of bias in studies	18	Present assessments of risk of bias for each included study.	n.a.
Results of individual studies	19	For all outcomes, present, for each study: (a) summary statistics for each group (where appropriate) and (b) an effect estimate and its precision (e.g., confidence/credible interval), ideally using structured tables or plots.	8–23
Results of syntheses	20a	For each synthesis, briefly summarise the characteristics and risk of bias among contributing studies.	8–23
	20b	Present results of all statistical syntheses conducted. If meta-analysis was done, present for each the summary estimate and its precision (e.g., confidence/credible interval) and measures of statistical heterogeneity. If comparing groups, describe the direction of the effect.	8–23
**DISCUSSION**			
Discussion	23a	Provide a general interpretation of the results in the context of other evidence.	23–24
	23b	Discuss any limitations of the evidence included in the review.	28
	23c	Discuss any limitations of the review processes used.	28
	23d	Discuss implications of the results for practice, policy, and future research.	23–28
**OTHER INFORMATION**			
Registration and protocol	24a	Provide registration information for the review, including register name and registration number, or state that the review was not registered.	3
	24b	Indicate where the review protocol can be accessed, or state that a protocol was not prepared.	3
	24c	Describe and explain any amendments to information provided at registration or in the protocol.	3
Support	25	Describe sources of financial or non-financial support for the review, and the role of the funders or sponsors in the review.	29
Competing interests	26	Declare any competing interests of review authors.	29
Availability of data, code and other materials	27	Report which of the following are publicly available and where they can be found: template data collection forms; data extracted from included studies; data used for all analyses; analytic code; any other materials used in the review.	n.a.

## References

[1] Pines J.M., Griffey R.T., Cone D.C. (2015). What We Have Learned From a Decade of ED Crowding Research. Acad. Emerg. Med..

[2] Hoot N.R., Aronsky D. (2008). Systematic Review of Emergency Department Crowding: Causes, Effects, and Solutions. Ann. Emerg. Med..

[3] Morley C., Unwin M., Peterson G.M., Stankovich J., Kinsman L. (2018). Emergency Department Crowding: A Systematic Review of Causes, Consequences and Solutions. PLoS ONE.

[4] Sun B.C., Hsia R.Y., Weiss R.E., Zingmond D., Liang L.-J., Han W., McCreath H., Asch S.M. (2013). Effect of Emergency Department Crowding on Outcomes of Admitted Patients. Ann. Emerg. Med..

[5] Carter E.J., Pouch S.M., Larson E.L. (2014). The Relationship Between Emergency Department Crowding and Patient Outcomes: A Systematic Review. J. Nurs. Scholarsh..

[6] Bernstein S.L., Aronsky D., Duseja R., Epstein S., Handel D., Hwang U., McCarthy M., McConnell K.J., Pines J.M., Rathlev N. (2009). The Effect of Emergency Department Crowding on Clinically Oriented Outcomes. Acad. Emerg. Med..

[7] Esteva A., Robicquet A., Ramsundar B., Kuleshov V., DePristo M., Chou K., Cui C., Corrado G., Thrun S., Dean J. (2019). A Guide to Deep Learning in Healthcare. Nat. Med..

[8] Alshiakh S.M., Alshamrani H.M., Ashari E.M., Sabgul A.A., Bamakhish N.F., Alyamani Y.K., Abuzaid F.A., Alhazmi F.S. (2025). Emergency Department Overcrowding And Its Impact On Patient Outcomes: A Systematic Review. Rev. Diabet. Stud..

[9] Alasmari A.M., Farooqi N.S., Alotaibi Y.A. (2024). Recent Trends in Crowd Management Using Deep Learning Techniques: A Systematic Literature Review. J. Umm Al-Qura Univ. Eng. Archit..

[10] Alishahi Tabriz A., Birken S.A., Shea C.M., Fried B.J., Viccellio P. (2019). What Is Full Capacity Protocol, and How Is It Implemented Successfully?. Implement. Sci. IS.

[11] American College of Emergency Physicians, Emergency Department Crowding Task Force (2008). Emergency Department Crowding: High-Impact Solutions.

[12] Pines J.M., Hilton J.A., Weber E.J., Alkemade A.J., Al Shabanah H., Anderson P.D., Bernhard M., Bertini A., Gries A., Ferrandiz S. (2011). International Perspectives on Emergency Department Crowding. Acad. Emerg. Med..

[13] Lee S.-Y., Chinnam R.B., Dalkiran E., Krupp S., Nauss M. (2020). Prediction of Emergency Department Patient Disposition Decision for Proactive Resource Allocation for Admission. Health Care Manag. Sci..

[14] McKenna P., Heslin S.M., Viccellio P., Mallon W.K., Hernandez C., Morley E.J. (2019). Emergency Department and Hospital Crowding: Causes, Consequences, and Cures. Clin. Exp. Emerg. Med..

[15] Salway R., Valenzuela R., Shoenberger J., Mallon W., Viccellio A. (2017). Emergency Department (ED) Overcrowding: Evidence-Based Answers to Frequently Asked Questions. Rev. Médica Clínica Las Condes.

[16] Hammer C., DePrez B., White J., Lewis L., Straughen S., Buchheit R. (2022). Enhancing Hospital-Wide Patient Flow to Reduce Emergency Department Crowding and Boarding. J. Emerg. Nurs..

[17] Marshall I.J., Wallace B.C. (2019). Toward Systematic Review Automation: A Practical Guide to Using Machine Learning Tools in Research Synthesis. Syst. Rev..

[18] Husein R.A., Aburajouh H., Catal C. (2025). Large Language Models for Code Completion: A Systematic Literature Review. Comput. Stand. Interfaces.

[19] Touvron H., Lavril T., Izacard G., Martinet X., Lachaux M.-A., Lacroix T., Rozière B., Goyal N., Hambro E., Azhar F. (2023). LLaMA: Open and Efficient Foundation Language Models. arXiv.

[20] A Survey on Evaluation of Large Language Models|ACM Transactions on Intelligent Systems and Technology. https://dl.acm.org/doi/10.1145/3641289.

[21] Page M.J., McKenzie J.E., Bossuyt P.M., Boutron I., Hoffmann T.C., Mulrow C.D., Shamseer L., Tetzlaff J.M., Akl E.A., Brennan S.E. (2021). The PRISMA 2020 Statement: An Updated Guideline for Reporting Systematic Reviews. BMJ.

[22] Covidence Systematic Review Software. http://www.Covidence.Org.

[23] Kadri F., Dairi A., Harrou F., Sun Y. (2022). Towards Accurate Prediction of Patient Length of Stay at Emergency Department: A GAN-Driven Deep Learning Framework. J. Ambient. Intell. Humaniz. Comput..

[24] Chen C.-H., Hsieh J.-G., Cheng S.-L., Lin Y.-L., Lin P.-H., Jeng J.-H. (2020). Early Short-Term Prediction of Emergency Department Length of Stay Using Natural Language Processing for Low-Acuity Outpatients. Am. J. Emerg. Med..

[25] Pak A., Gannon B., Staib A. (2021). Predicting Waiting Time to Treatment for Emergency Department Patients. Int. J. Med. Inf..

[26] Chrusciel J., Girardon F., Roquette L., Laplanche D., Duclos A., Sanchez S. (2021). The Prediction of Hospital Length of Stay Using Unstructured Data. BMC Med. Inform. Decis. Mak..

[27] Seo H., Ahn I., Gwon H., Kang H.J., Kim Y., Cho H.N., Choi H., Kim M., Han J., Kee G. (2024). Prediction of Hospitalization and Waiting Time within 24 Hours of Emergency Department Patients with Unstructured Text Data. Health Care Manag. Sci..

[28] Zeleke A.J., Palumbo P., Tubertini P., Miglio R., Chiari L. (2023). Machine Learning-Based Prediction of Hospital Prolonged Length of Stay Admission at Emergency Department: A Gradient Boosting Algorithm Analysis. Front. Artif. Intell..

[29] Li M., Vanberkel P., Zhong X. (2022). Predicting Ambulance Offload Delay Using a Hybrid Decision Tree Model. Socio-Econ. Plan. Sci..

[30] Benevento E., Aloini D., Squicciarini N. (2023). Towards a Real-Time Prediction of Waiting Times in Emergency Departments: A Comparative Analysis of Machine Learning Techniques. Int. J. Forecast..

[31] Gurazada S.G., Gao S.C., Burstein F., Buntine P. (2022). Predicting Patient Length of Stay in Australian Emergency Departments Using Data Mining. Sensors.

[32] Smith A.J., Patterson B.W., Pulia M.S., Mayer J., Schwei R.J., Nagarajan R., Liao F., Shah M.N., Boutilier J.J. (2023). Multisite Evaluation of Prediction Models for Emergency Department Crowding before and during the COVID-19 Pandemic. J. Am. Med. Inform. Assoc. JAMIA.

[33] Tomović S. (2021). Patient Length of Stay Analysis with Machine Learning Algorithms. Comput. Sci. Inf. Syst..

[34] Wang G. (2022). The Effect of Medicaid Expansion on Wait Time in the Emergency Department. Manag. Sci..

[35] Arora S., Taylor J.W., Mak H.-Y. (2023). Probabilistic Forecasting of Patient Waiting Times in an Emergency Department. Manuf. Serv. Oper. Manag..

[36] Giunta D.H., Thomas D.S., Bustamante L., Ratti M.F.G., Martinez B.J. (2025). Development and Validation of a Multivariable Predictive Model for Emergency Department Overcrowding Based on the National Emergency Department Overcrowding Study (NEDOCS) Score. Intern. Emerg. Med..

[37] Aldhoayan M.D., Al Harbi A.S., Arajhi K., Abduljawad J. (2022). Statistical and Machine Learning Analysis of Non-Clinical Factors Impacting Emergency Room Delays. Inform. Med. Unlocked.

[38] Tuominen J., Pulkkinen E., Peltonen J., Kanniainen J., Oksala N., Palomäki A., Roine A. (2024). Forecasting Emergency Department Occupancy with Advanced Machine Learning Models and Multivariable Input. Int. J. Forecast..

[39] Aziz W., Nicalaou A., Stylianides C., Panayides A., Kakas A., Kyriacou E., Pattichis C. (2024). Emergency Department Length of Stay Classification Based on Ensemble Methods and Rule Extraction. Stud. Health Technol. Inform..

[40] Sulaiman W.A., Stylianides C., Nikolaou A., Antoniou Z., Constantinou I., Palazis L., Vavlitou A., Kyprianou T., Kyriacou E., Kakas A. (2025). Leveraging Machine Learning and Rule Extraction for Enhanced Transparency in Emergency Department Length of Stay Prediction. Front. Digit. Health.

[41] Cheng N., Kuo A. (2020). Using Long Short-Term Memory (LSTM) Neural Networks to Predict Emergency Department Wait Time. Stud. Health Technol. Inform..

[42] Arnaud E., Elbattah M., Ammirati C., Dequen G., Ghazali D.A. (2022). Use of Artificial Intelligence to Manage Patient Flow in Emergency Department during the COVID-19 Pandemic: A Prospective, Single-Center Study. Int. J. Environ. Res. Public Health.

[43] Moya-Carvajal J., Pérez-Galarce F., Taramasco C., Astudillo C.A., Candia-Véjar A. (2023). ML Models for Severity Classification and Length-of-Stay Forecasting in Emergency Units. Expert Syst. Appl..

[44] Cheng Q., Argon N.T., Evans C.S., Liu Y., Platts-Mills T.F., Ziya S. (2021). Forecasting Emergency Department Hourly Occupancy Using Time Series Analysis. Am. J. Emerg. Med..

[45] Parnass G., Levtzion-Korach O., Peres R., Assaf M. (2023). Estimating Emergency Department Crowding with Stochastic Population Models. PLoS ONE.

[46] Wartelle A., Mourad-Chehade F., Yalaoui F., Laplanche D., Sanchez S. (2022). Analysis of Saturation in the Emergency Department: A Data-Driven Queuing Model Using Machine Learning. Stud. Health Technol. Inform..

[47] Etu E.-E., Monplaisir L., Arslanturk S., Masoud S., Aguwa C., Markevych I., Miller J. (2022). Prediction of Length of Stay in the Emergency Department for COVID-19 Patients: A Machine Learning Approach. IEEE Access.

[48] Ataman M.G., Sarıyer G. (2021). Predicting Waiting and Treatment Times in Emergency Departments Using Ordinal Logistic Regression Models. Am. J. Emerg. Med..

[49] Kuo Y.-H., Chan N.B., Leung J.M.Y., Meng H., So A.M.-C., Tsoi K.K.F., Graham C.A. (2020). An Integrated Approach of Machine Learning and Systems Thinking for Waiting Time Prediction in an Emergency Department. Int. J. Med. Inf..

[50] Naemi A., Schmidt T., Mansourvar M., Ebrahimi A., Wiil U.K. (2021). Quantifying the Impact of Addressing Data Challenges in Prediction of Length of Stay. BMC Med. Inform. Decis. Mak..

[51] Shbool M.A., Arabeyyat O.S., Al-Bazi A., Al-Hyari A., Salem A., Abu-Hmaid T., Ali M. (2023). Machine Learning Approaches to Predict Patient’s Length of Stay in Emergency Department. Appl. Comput. Intell. Soft Comput..

[52] Rahman M.A., Honan B., Glanville T., Hough P., Walker K. (2020). Using Data Mining to Predict Emergency Department Length of Stay Greater than 4 Hours: Derivation and Single-Site Validation of a Decision Tree Algorithm. Emerg. Med. Australas..

[53] Haraldsson T., Marzano L., Krishna H., Lethval S., Falk N., Bodeby P., Raghothama J., Meijer S., Darwich A.S. (2024). Exploring Hospital Overcrowding with an Explainable Time-to-Event Machine Learning Approach. Stud. Health Technol. Inform..

[54] Ricciardi C., Marino M.R., Trunfio T.A., Majolo M., Romano M., Amato F., Improta G. (2024). Evaluation of Different Machine Learning Algorithms for Predicting the Length of Stay in the Emergency Departments: A Single-Centre Study. Front. Digit. Health.

